# Modelling to Quantify the Likelihood that Local Elimination of Transmission has Occurred Using Routine *Gambiense* Human African Trypanosomiasis Surveillance Data

**DOI:** 10.1093/cid/ciab190

**Published:** 2021-06-14

**Authors:** Christopher N Davis, María Soledad Castaño, Maryam Aliee, Swati Patel, Erick Mwamba Miaka, Matt J Keeling, Simon E F Spencer, Nakul Chitnis, Kat S Rock

**Affiliations:** 1 Mathematics Institute, University of Warwick, Coventry, United Kingdom; 2 Zeeman Institute for Systems Biology and Infectious Disease Epidemiology Research (SBIDER), University of Warwick, Coventry, United Kingdom; 3 Department of Epidemiology and Public Health, Swiss Tropical and Public Health Institute, Basel, Switzerland; 4 University of Basel, Basel, Switzerland; 5 Department of Statistics, University of Warwick, Coventry, United Kingdom; 6 Programme National de Lutte contre la Trypanosomiase Humaine Africaine, Kinshasa, the Democratic Republic of the Congo; 7 School of Life Science, University of Warwick, Coventry, United Kingdom

**Keywords:** *gambiense* human African trypanosomiasis (gHAT), modeling, elimination of transmission, surveillance

## Abstract

**Background:**

The gambiense human African trypanosomiasis (gHAT) elimination programme in the Democratic Republic of Congo (DRC) routinely collects case data through passive surveillance and active screening, with several regions reporting no cases for several years, despite being endemic in the early 2000s.

**Methods:**

We use mathematical models fitted to longitudinal data to estimate the probability that selected administrative regions have already achieved elimination of transmission (EOT) of gHAT. We examine the impact of active screening coverage on the certainty of model estimates for transmission and therefore the role of screening in the measurement of EOT.

**Results:**

In 3 example health zones of Sud-Ubangi province, we find there is a moderate (>40%) probability that EOT has been achieved by 2018, based on 2000–2016 data. Budjala and Mbaya reported zero cases during 2017–18, and this further increases our respective estimates to 99.9% and 99.6% (model S) and to 87.3% and 92.1% (model W). Bominenge had recent case reporting, however, that if zero cases were found in 2021, it would substantially raise our certainty that EOT has been met there (99.0% for model S and 88.5% for model W); this could be higher with 50% coverage screening that year (99.1% for model S and 94.0% for model W).

**Conclusions:**

We demonstrate how routine surveillance data coupled with mechanistic modeling can estimate the likelihood that EOT has already been achieved. Such quantitative assessment will become increasingly important for measuring local achievement of EOT as 2030 approaches.

The decline in reported cases of the parasitic infection *gambiense* human African trypanosomiasis (gHAT) in the Democratic Republic of Congo (DRC) is a strong indicator of the progress toward elimination objectives. The World Health Organization (WHO) established goals for elimination in 2012: elimination of gHAT as a public health problem (EPHP) by 2020, and elimination of transmission (EOT) by 2030 [1, 2]. In the DRC, which has consistently been the country with the highest global gHAT burden (>69% in each of the last 10 years), there were 26 318 cases at the peak of infection in 1998; however by 2019, just 604 cases of gHAT infection were reported [3].

Cases of gHAT, which are commonly fatal when untreated [4], are typically reported by routine surveillance, which comprises active screening and passive surveillance. There is no prophylactic available, and mass drug administration is not viable with currently approved treatments; as a result, control of the infection is predominantly through a series of serological testing, parasitological confirmation, and then treatment. In active screening, at-risk settlements are targeted for the mass screening of all residents [2]. Passive surveillance is conducted in fixed health centers with available gHAT diagnostic tools, where patients with symptoms can self-present and be tested for infection. These surveillance strategies, designed to deliver effective control for gHAT, also double to provide valuable information on monitoring and evaluation of the gHAT situation across endemic settings.

With a reduction in the number of cases reported, some regions of the DRC, which were previously endemic at the beginning of the twenty-first century, are now reporting no new cases in multiple years of annual reporting. This is true in several health zones of the former Equateur province (now consisting of the 5 provinces Nord-Ubangi, Sud-Ubangi, Equateur, Mongala, and Tshuapa). The former Equateur province had the highest burden of gHAT in the DRC in 2000 but, following extensive active screening campaigns, now reports far fewer infections [1, 5, 6]. This low reporting introduces the possibility that local EOT may have already been achieved in some health zones—administrative regions made up of approximately 100 000 inhabitants. However, with intensified disease management infections, such as gHAT, the interplay between surveillance and control is complex; gHAT active screening will reduce in regions with little or no reporting, but this in turn could lead to less chance of detecting remaining infection if it is stopped too early.

Current World Health Organization (WHO) guidance suggests cessation of active screening in a village once 3 years have been observed with no case reporting [2], which is thought to give reasonable probabilities that EOT is met in the village (>90% probability for villages with fewer than 2000 inhabitants when active screening coverages are over 20% in the village [7]) and be a cost-effective use of resources [8]. However, it is less clear how diminishing screening over time across health zones impacts our ability to assess whether there is ongoing transmission.

Several modeling studies have addressed EOT for gHAT [9]. The expected time to EOT has been estimated in both specific regions [10] and across each of the health zones of DRC reporting gHAT data in the last 20 years [11]. The value of screening information for EOT has also been considered [7, 10] and the cost-effectiveness of active screening in the DRC assessed [8, 12]. However, there has been no assessment of how available data can be used to estimate the probability that EOT has already occurred.

In the present study, we utilize 2 previously developed models of gHAT transmission with parameters matched to the screening and case data from 3 health zones in the Sud-Ubangi province of the DRC: Bominenge, Budjala, and Mbaya. These health zones have all had zero or low numbers of reported gHAT cases in the last decade, despite varying active screening efforts. We estimate the changing transmission over time and thereby calculate the probability that these health zones have already achieved EOT. We also project forward to determine the probability of EOT up until 2040. Due to a reduction in active screening in these health zones, we examine how increasingly limited data impact the certainty of our model estimates and highlight that active screening is a key tool in measuring elimination.

## METHODS

### Description of Data

Data on active screening and passive surveillance is made available through the WHO HAT Atlas [1, 5, 6]. In this study, we use data from 2000 to 2018 of the annual screening numbers and the number of confirmed cases in active screening and passive surveillance for 3 health zones (Bominenge, Budjala, and Mbaya). In addition to case numbers, the infection stage of most cases is known from 2015 onward, and this provides some information about the duration of infection in these patients. Stages of gHAT are defined by the point at which parasites cross the blood-brain barrier, and stage 2 (late stage) is diagnosed through detection of trypanosomes in the cerebrospinal fluid (CSF) or an elevated white blood cell (WBC) count in the CSF (>5 WBC/µL). Traditionally, different treatments were required for the different stages, which is why lumbar puncture was routinely performed. The new treatment—fexinidazole—removes the need for staging in patients without severe symptoms; however, this was not approved for use in DRC until after the data period [13].

From the selected health zones (see Table 1), Bominenge has had the best recent active screening coverage and, despite this relatively extensive case-finding effort, only a small number of reported cases. Budjala has had a low active screening coverage, and only one reported case in the last 5 years. Mbaya has had a very low active screening coverage and last reported a single case in 2015. Thus, for all 3 health zones, the small case numbers suggest that transmission may have already been halted in these regions.

**Table 1.** Active Screening Coverage and New Cases in the Last Five years of Data for Our Three Example Health Zones in Sud-Ubangi Province

Health Zone	Population Size (2015)	Number of People in Active Screening (2014–2018)	New Cases Detected (2014–2018)
Bominenge	156 827	67 916 (12 576, 22 423, 32 917, 11 002, 20 650 in each year 2014–2018, respectively)	12 (including 2 in 2018)
Budjala	129 539	2713 (all in 2016)	1 (in 2016)
Mbaya	66 457	0	1 (in 2015)

The population size of each health zone is reported in Table 1, with Mbaya being substantially smaller than Budjala and Bominenge. We assume a 3% annual population growth rate in these health zones for comparison with the observed data.

### Modeling Approach

To estimate the likelihood of having achieved EOT in the health zones, we define mechanistic models of gHAT infection adapted from previous work (originally presented in Rock et al [14] and Stone et al [15] and adapted in numerous ways since then; see supplementary material for detailed information). The models capture the underlying infection dynamics, which are defined in a stochastic framework because we need to account for the chance events of infection, which will be amplified due to the low numbers in the regions [10]. Furthermore, the stochastic framework means we can directly measure EOT in the model outcomes, which cannot be done without defining a threshold of elimination if using a deterministic framework.

We use 2 independent models developed by different teams with different structures and assumptions (model W and model S) to provide a more complete picture on the uncertainty of our predictions and greater confidence when the models provide aligned results. Both models use parameters matched to the data for 2000–2016 from the 3 health zones using a deterministic variant of their model. For each health zone, the dynamics are averaged over 200 000 stochastic simulations, whereby 1000 realizations are executed for each of 200 parameter sets, taken from the posterior of the deterministic model fits, to account for any parameter uncertainty. A full description of each model and details of the model fitting processes are given in the supplementary material.

We calculate the probability of EOT at each time point as the proportion of stochastic simulations that result in EOT (see supplementary material for full definition). In conducting the model realizations, simulated active screening events use the same coverage that appears in the data for the known period (2000–2018), with unknown and future screening coverages taken as the mean value from the last 5 years of data (2014–2018); passive surveillance is assumed to continue from 2019 at the same level as 2000–2018 for model W and at the highest value from the data period for model S. The coronavirus disease 2019 (COVID-19) pandemic caused an interruption of active screening activities in the DRC in 2020 [16, 17]. However, because the active screening coverage is already considered very low in these health zones, we do not explicitly consider this impact here; we assume passive surveillance was not altered.

Finally, we also consider what proportion of our realizations have zero case observations in various years. For example, although the model fitting only used data for 2000–2016, we now know that Budjala and Mbaya reported zero cases in both 2017 and 2018, so we look to see how, using only realizations that match this reporting, this data changes the probability estimates for EOT. Similarly, we consider what would happen to the probability of achieving EOT by 2021 if either the mean active screening was conducted and no cases were reported in each health zone, or if a very large active screening with 50% coverage was performed, also with no cases reported. Under model S, perfect specificity was assumed for this active screening, but not in other years. This enables us to investigate whether there is value in using a one-off, large active screening for monitoring and evaluation purposes.

## RESULTS

**Figure 1.**
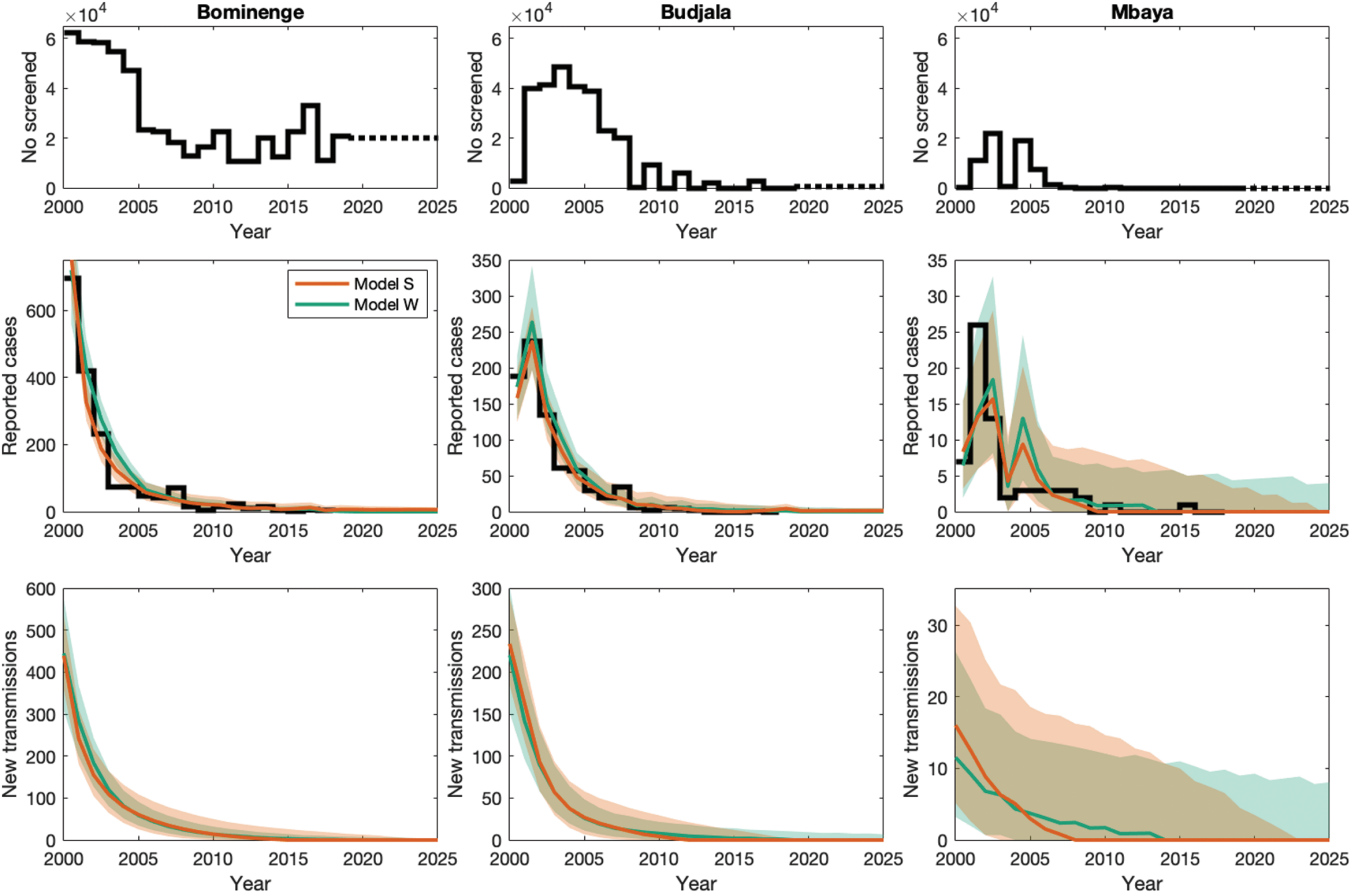
Case reporting and inferred infection dynamics by the 2 models in 3 health zones for Sud-Ubangi province, DRC. The first row shows the number of people screened in each year in each health zone, with dashed lines representing our assumed future active screening coverage. The second row shows the total reported case data as a black solid line and the model fits as colored lines (median) and shaded area (95% credible and prediction intervals). The last row shows our estimated number of new infections in humans (transmission) over time. Model S is orange, and model W is green. Individual model results showing mean values can be found in the SI.

Simulations of the 2 models in the 3 health zones are shown in Figure 1. These simulations used model parameters obtained from fitting to 2000–2016 data and used known screening data for 2000–2018. From 2019 the simulations presented here use continued mean active screening each year. In Bominenge and Budjala, the models produce relatively tight credible intervals for case reporting and new infections due to the high case numbers in the early 2000s and the high coverage active screening, both of which reduce our uncertainty. During 2000–2016, the 2 models’ credible intervals are largely overlapping for each of these health zones. In Mbaya, there has not been very high case reporting nor screening during 2000–2016, and uncertainty in the inferred transmission and predicted future reporting is larger. Model W has a wide tail on new infections for Mbaya compared to model S and is, in part, explained by model S assuming improvement in passive surveillance during the data period, whereas model W assumes constant passive detection rates throughout. A similar long-tailed effect in Budjala is seen under model W.

In Bominenge and Budjala, both models estimate that new infections have fallen more than 99% during the data period (2000–2018). In Mbaya, the percentage decrease is smaller at 91% (model W) and 97% (model S), linked to less active screening and the large challenge of moving from low to zero infections, with small and hard-to-identify pockets of infection remaining.

**Figure 2.**
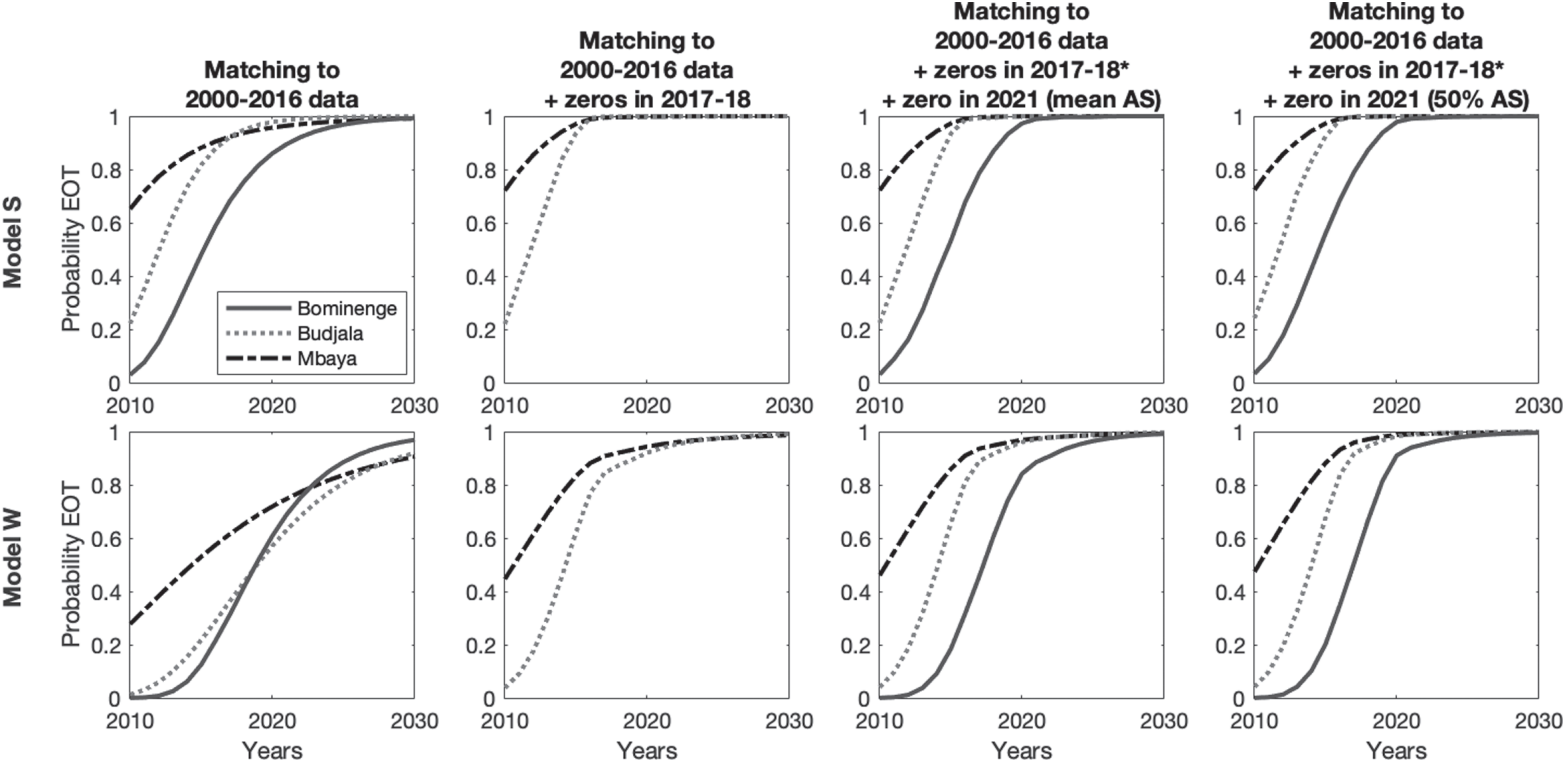
Probability of EOT by year for each of the models. The top row is the results for model S and the bottom row for model W. Each column represents our results based on model fitting to data for the period 2000–2016 and using known screening coverage for 2017 and 2018. For 2019 onward, these are predictions assuming continuation of the mean active screening coverage (based on 2014–18 coverage) and passive surveillance. In the second to fourth columns, we only show the subset of results that also meet additional criteria. The second column shows the probability of EOT for those simulations that have zero case reporting in 2017 and 2018 in Budjala and Mbaya (matching the reported data for those years). In the third and fourth columns, we show the subset of results if zero cases are observed in 2021 under mean active screening (third column) or a 50% coverage screen (fourth column); we allow cases to be detected in Bominenge during 2017–18 but not in Budjala or Mbaya. Abbreviation: EOT, elimination of transmission.

Overall, using the results from all model fits to data from 2000 to 2016, model S is more optimistic that the 3 health zones may have already achieved EOT. However, both models find there is a wider distribution of predicted elimination years for Mbaya, shown by the shallower slope in the probability of EOT over time. In Mbaya, only one case has been reported since 2009, which, on the surface, may suggest EOT is likely to have been achieved. This corresponds well with modelling predictions of a 72% (model W) and 96% (model S) probability of EOT by 2020 (Figure 2, first column). However, this probability is lower than the other health zones for future times as the very low levels of active screening provide fewer data, so there is much more uncertainty in these predictions.

Using all simulation results, we predict a moderate (>57%) probability of EOT in the present day (2020) in all health zones and under both models (Figure 2, first column). By selecting subsets of our simulations that match the zero case reporting that occurred in 2017 and 2018 for Budjala and Mbaya, we see that the probability that EOT has already been met increases for both models and in both health zones. These additional 2 years of zeros raise the probability more under model W (57% to 92% for Budjala and 72% to 94% in Mbaya) compared to model S (98% to >99% for Budjala and 98% to >99% in Mbaya). In total, 33% and 0.3% of simulations match the 2017/18 zero case reporting in Budjala and 62% and 90% match it in Mbaya (models W and S, respectively).

Bominenge has had low level case reporting in 2017 and 2018; however, if there were no cases reported in 2021, there would be an estimated 99% probability of having met EOT under model S and 89% under model W. Without this information, the models currently have lower certainty in EOT: 86% in 2020 for model S and 61% in model W. It is noted that the mean coverage of active screening for 2014–2018 is very low in all the health zones (12% for Bominenge, and virtually no screening in Budjala and Mbaya); however, by doing a single large screen covering 50% of the population in 2021, this could further raise the estimated probability that EOT had been achieved to 94% if zero cases were found under model W; model S finds this still results in a >99% probability (virtually no change).

## Discussion

As infections fall across gHAT endemic areas, more locations will achieve local EOT. Cases provide an indication of the underlying transmission; however, we cannot directly equate cases with transmission or remaining infection. The number of cases includes time lags in reporting infections, such that the last infection event could occur years before the last remaining infected person is found and treated or dies. In contrast, insufficient active screening and underreporting may present transmission as much lower than in reality [9]. Therefore, mathematical modeling is useful in untangling the relationship between case data and true infections.

In this study, we have considered health zones of the Sud-Ubangi province that have seen a large decline in reported cases, where the data indicate EOT may have been met already or would be expected soon. Indeed, we have estimated moderate probabilities of EOT by 2016 for all 3 health zones (Figure 2).

The future projection of the probability of EOT for Mbaya provides lower estimates than the other health zones, as the very low active screening coverage provides less surveillance information and so greater uncertainty for predictions (Figure 2, first column). This underlines the importance of a passive surveillance system that can provide data on the testing of individuals within health facilities. Additional information, that no cases were reported in 2017–18, provides a greater certainty of EOT for future times (Figure 2, second column), which is further increased if another active screening reported no cases (Figure 2, third and fourth columns).

For Budjala and Bominenge, we have estimated high probabilities of EOT within the next 5 years, assuming active screening will continue with similar coverage. Past high coverage of active screening (eg, up to 62% in Bominenge and 53% in Budjala) enables better model estimation of transmission patterns in the health zone. Maintaining a high active screening coverage can both reduce any remaining transmission, by the detection and treatment of infection, and provides the surveillance mechanism required to have confidence that EOT has been reached. A single year of high-coverage active screening resulting in no new case detections would be very informative in establishing EOT in locations where there was lower certainty based on past data.

All predictions are under the assumption that, despite different levels of active screening, the efficacy of future passive surveillance will remain as good as during the data period. Passive surveillance is critical to be maintained to provide the support for people not tested in active screening, particularly in these low transmission areas where, without it, there would be no measure of the infection level [18]. Health facilities in these regions need to continue to be equipped with diagnostics, such that gHAT can be rapidly identified and diagnosed where it occurs, to reduce the probability of future transmission.

This study did not take into account the cost of gHAT interventions. Therefore, although we strongly advocate for bolstered active screening in regions where it has been limited recently, if the goal is to improve information, this is not necessarily a cost-effective use of resources in terms of averting morbidity or mortality. The value of this information in a cost framework is beyond the scope of this analysis but would be an interesting avenue for future work, in which the challenges of optimizing limited resources in an end-game setting could be explored.

Movement of people, asymptomatic infections in humans, and the potential for animal reservoirs to reintroduce infection [19, 20] provide additional challenges in estimating EOT. Our models make the assumption that animals do not contribute to the gHAT transmission process in humans, because their role in this is unclear [21]. Furthermore, we do not account for a reservoir of asymptomatic humans that are infected but are difficult to detect due to the parasites surviving in the skin, rather than the blood. These individuals are known to exist, but the extent and consequence for elimination campaigns remains unknown [22]. Therefore, our predictions for the year of EOT could be altered if these types of undetected transmission occur frequently.

## Conclusions

Historically good coverage in active screening has been shown to be effective in reducing gHAT infection, but maintaining this coverage can provide an accurate measure on the probability EOT has been met in health zones close to this goal. Passive surveillance remains a vital control mechanism, but broader screening can help increase the certainty in measurement of EOT. Active screening in previously endemic areas could therefore be useful in certifying regional EOT. Modeling can be used to identify regions for which this could provide improved certainty, and those where further active screening is unlikely to be required.

In this study the health zones of Bominenge, Budjala, and Mbaya in the Sud-Ubangi province were found very likely to meet the EOT goal by 2030; indeed, Budjala and Mbaya were found to have already met this goal by 2020 with >92% probability. Bominenge, in particular, has lower certainty that the goal has been already met (61% under model W and 86% under model S); however, a one-off year of high coverage active screening could provide valuable information to better inform this. As we approach 2030, quantitative evaluation of gHAT data will be key to safe cessation of activities and reducing the risk of recrudescence in areas believed to have no remaining transmission.

## Supplementary Data

Supplementary materials are available at *Clinical Infectious Diseases* online. Consisting of data provided by the authors to benefit the reader, the posted materials are not copyedited and are the sole responsibility of the authors, so questions or comments should be addressed to the corresponding author.

## Supplementary Material

ciab190_suppl_Supplementary-Material

ciab190_suppl_Supplementary-Data

## Notes

***Acknowledgments.*** We thank the World Health Organization human African trypanosomiasis (HAT) team for facilitating access to the HAT Atlas data [1] and for helpful discussion and comments on this manuscript and PNLTHA of DRC for the original data collection.

***Supplement sponsorship.*** This article appears as part of the supplement “Sustainable control of neglected tropical diseases – surveillance and diagnostics”, sponsored by the NTD Modelling Consortium.

***Disclaimer.*** The funders had no role in study design, data collection and analysis, decision to publish, or preparation of the manuscript.

***Funding.*** This work was supported by the Bill and Melinda Gates Foundation (www.gatesfoundation.org) through the NTD Modeling Consortium (grant number (OPP1184344) and (OPP1177824)) (C. N. D., M. A., M. S. C, S. P., K. S. R., S. E. F. S, N. C., and M. J. K.) and through the Human African Trypanosomiasis Modeling and Economic Predictions for Policy (HAT MEPP) project (grant number OPP1177824) (K. S. R. and M. J. K.).

***Potential Conflicts.*** E. M. reports grants from Directorate-general Development Cooperation and Humanitarian Aid. E. M. reports that the national sleeping sickness programme is part of the Ministry of Health in DRC. All other authors report no potential conflicts. All authors have submitted the ICMJE Form for Disclosure of Potential Conflicts of Interest. Conflicts that the editors consider relevant to the content of the manuscript have been disclosed.
