# Supplementary material for: Modelling to Quantify the Likelihood that Local Elimination of Transmission has Occurred Using Routine Gambiense Human African Trypanosomiasis Surveillance Data
Source: Clin Infect Dis. 2021 Jun 14;72(Suppl 3):S146–51. doi: 10.1093/cid/ciab190 (PMC8201550; doi:10.1093/cid/ciab190)
Supplement: ciab190_suppl_Supplementary-Material [file ciab190_suppl_supplementary-material.pdf]

# Supplementary Information
# Modelling to quantify the likelihood that local elimination of transmission has occurred using routine *gambiense* human African trypanosomiasis surveillance data

Christopher N Davis[1,2,*], M Soledad Castaño[3,4,*], Maryam Aliee[1,2,*], Swati Patel[2,5], Erick Mwamba Miaka[6], Matt J Keeling[1,2,7], Simon E F Spencer[2,5], Nakul Chitnis[3,4,**], Kat S Rock[1,2,**]

1 Mathematics Institute, University of Warwick, Coventry, United Kingdom
2 Zeeman Institute for Systems Biology and Infectious Disease Epidemiology Research (SBIDER), University of Warwick, Coventry, United Kingdom
3 Department of Epidemiology and Public Health, Swiss Tropical and Public Health Institute, Basel, Switzerland
4 University of Basel, Basel, Switzerland
5 Department of Statistics, The University of Warwick, Coventry, United Kingdom
6 Programme National de Lutte contre la Trypanosomiase Humaine Africaine, Kinshasa, the Democratic Republic of the Congo
7 School of Life Science, University of Warwick, Coventry, United Kingdom
∗Authors contributed equally
∗∗Authors contributed equally

## 1 Data

Data used for the fitting of Model W and Model S were extracted from the WHO HAT Atlas by using geolocation information (where available) and following the detailed algorithm laid out in the Supplementary Information of Crump *et al.* [1]. We use all data for each health zone in the years 2000–2016. Additional data for 2017 and 2018, extracted using health zone names from an updated version of the HAT Atlas, was used to match simulation results in Figure 2. The data for screening in 2017 and 2018 as also used as part of model projections. The 2017 and 2018 data had no impact on the fit of either model.

## 2 Model W

Model W has been described many times elsewhere [1–12] and so the text the follows is very similar to descriptions found in other articles. Model W has evolved since its first using in Rock *et al.* [2] based on fitting to new data sets, improved fitting methods, and new information about implementation of HAT strategies, therefore we describe the model used in the present study in full to avoid any confusion with slightly different, earlier versions.

### 2.1 Description

The Model W presented in this study is a stochastic version of the ODE model presented in Rock *et al.* [2] and Crump *et al.* [1] and similar to the stochastic model in Castaño *et al.* [8]. The original model describes dynamics of gHAT transmission explicitly considering compartments of humans and tsetse. Figure 1 shows a schematic description of HAT dynamics in this model. Humans can be exposed and subsequently infectious by a bite of an infectious tsetse. They progress through different stages of the infection (Stage 1 and Stage 2) with different rates ($\sigma_H$ and $\phi_H$ respectively). On the other side, tsetse vectors can become exposed and subsequently infectious if

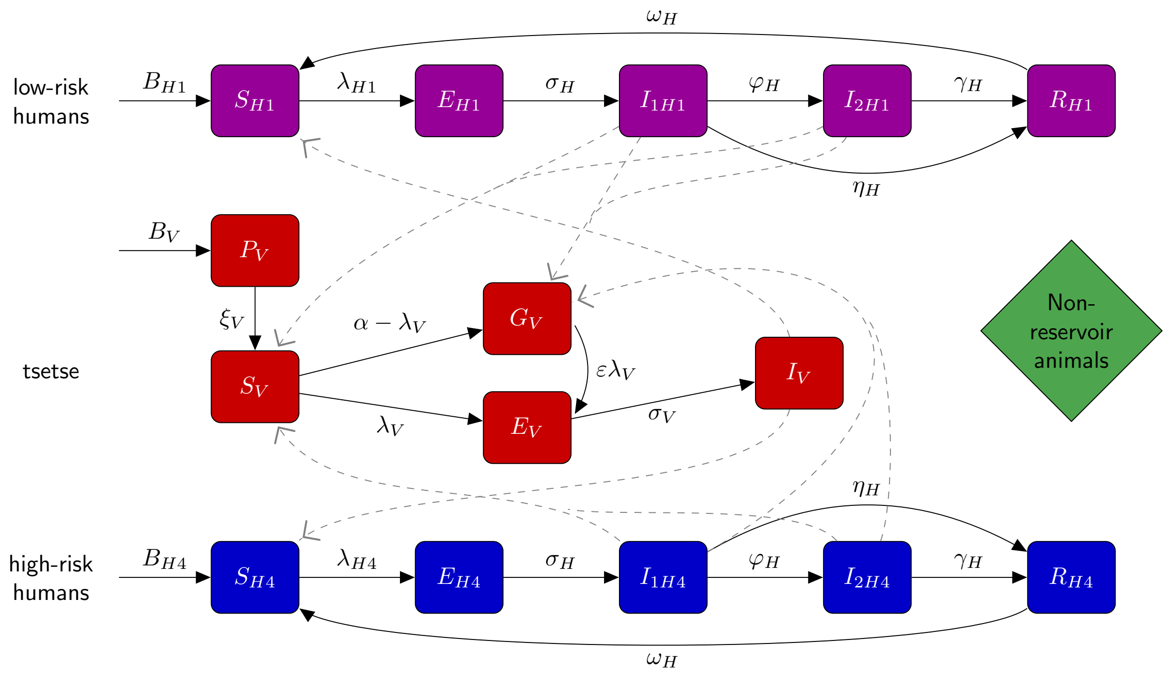


Figure 1: **Schematic of gHAT infection dynamics used in Model W.** This multi-host model of HAT takes into account high- and low-risk groups of humans and their interactions with tsetse vectors. Each group consists of different compartments: Susceptible humans $S_{Hi}$ can become exposed on a bite of an infectious tsetse. Exposed people $E_{Hi}$ progress to become the Stage $1$ infected people and eventually Stage $2$ (if not detected in active screening), and once treated they recover by hospitalization $R_{Hi}$. Active screening can accelerate treatment rate of infected people. Here we assume high-risk group does not participate in active screening. By biting an infectious person, tsetse can become exposed and subsequently infectious, $E_V$ and $I_V$. $G_V$ represents the tsetse population not exposed to *Trypanosoma brucei gambiense* in the first blood-meal and are therefore less susceptible in the following meals. Rates are shown by Greek letters associated with arrows. Animal reservoir is not considered. This figure is taken from [1] and adapted from the original model schematic [2].

they bite an infectious human. Infected people may be detected by passive and active screening (more details in 2.2), followed by hospitalisation and recovery. Here, we consider a version of the model where humans are partitioned into two compartments of (i) low-risk and participating in the active screening, and (ii) high-risk and non-participating in active screening. We assume there are no animal reservoirs although animals receive some proportion of tsetse bites. For simplicity, we assume the total population of humans to be constant, however, we take into account growth of population (3%) for comparison to the observed data (more details in Section 2.2).

In this stochastic model, individual humans are assigned to different compartments associated with infection/disease status and can transition between them. We describe system dynamics by random events captured by a tau-leap approximation. Table 1 explains different events and the corresponding rates that lead to one person transitioning from one compartment to another one. Within this framework, the number of events happening in a time interval $\tau$ is chosen randomly from a Poisson distribution with the mean equal to the event rate multiplied by $\tau$. We use time interval of a day in the tau-leaping algorithm that is shown to be a sensible choice for gHAT dynamics [7].

To avoid the need to disaggregate the parameter $m_{eff}$ — which was used to non-dimensionalise the previous ODE system — into its component parts ($p_H$ and $N_V/N_H$), we keep vector dynamics the same as the original

model, described by a set of ODEs:

$$
\begin{aligned}
\frac{dS_V}{dt} &= \mu_V N_H - \alpha S_V - \mu_V S_V \\
\frac{dE_{1V}}{dt} &= \alpha p_V (f_{H1}\frac{I_{1H1}+I_{2H1}}{N_{H1}} + f_{H2}\frac{I_{1H2}+I_{2H2}}{N_{H2}})(S_V + \varepsilon G_V) - (3\sigma_V + \mu_V)E_{1V} \\
\frac{dE_{2V}}{dt} &= 3\sigma_V E_{1V} - (3\sigma_V + \mu_V)E_{2V} \\
\frac{dE_{3V}}{dt} &= 3\sigma_V E_{2V} - (3\sigma_V + \mu_V)E_{3V} \\
\frac{dI_V}{dt} &= 3\sigma_V E_{3V} - \mu_V I_V \\
\frac{dG_V}{dt} &= \alpha \left(1 - p_V \left(f_{H1}\frac{I_{1H1}+I_{2H1}}{N_{H1}} + f_{H2}\frac{I_{1H2}+I_{2H2}}{N_{H2}}\right)\right) S_V \\
&- \alpha p_v \left(f_{H1}\frac{I_{1H1}+I_{2H1}}{N_{H1}} + f_{H2}\frac{I_{1H2}+I_{2H2}}{N_{H2}}\right) \varepsilon G_V - \mu_V G_V. \qquad (1)
\end{aligned}
$$

This is a legitimate assumption due to the high population of vectors and their short life cycle compared to humans. We solve these equations with the help of Runge-Kutta methods.

## 2.2 Screening

This model accounts for the possibility of detecting of infected humans through passive and active screening. Passive screening describes potential visits of people to fixed medical centers for testing. In the stochastic version, we identify the number of people participating in passive screening by a tau-leaping approximation with rates proportional to $\eta_H$ and $\gamma_H$ corresponding to the first and second stages of the disease (Table 1).

Before 1998 (pre-active screening) it was assumed that passive detection was less effective than after activities began, and only so identified Stage 2 individuals at a rate $\gamma_{H0}$, which is smaller than the Stage 2 passive detection rate from 1998 onwards, $\gamma_H$.

Unlike previous modelling work using gHAT data from former Bandundu province [1, 8], there is a no strong signal from epidemiological staging data that passive screening has improved during the time period from 2000–2016 in the health zones of Bominenge, Budjala and Mbaya. Therefore, we keep the passive detection rates constant after 1998.

In our current approach, active screening is considered to be a random procedure to detect infected humans within the people participating voluntarily. The number of people picked up from each compartment is given by a random number chosen from a binomial distribution. Similar to the previous models, we allow for the imperfect nature of the tests by considering sensitivity of tests to detect true cases and specificity to observe false positive cases. Specificity is set to one after 2018 due to improvement in confirmatory quality control [1].

Using a similar approach to the previous ODE models, in our model simulations here, we consider the same level of screening as reported between 2000–2018. For before 2000, as in many of the previous published studies using this model, we assume that active screening began in 1998 and achieved the same number of people screened as in 2000 (the first year of data). After 2018, we use the average and maximum percentage of screened people between 2014–2018 in different strategies.

## 2.3 Parameter values

As in previous versions of Model W [1–3, 6–10], some parameters with estimates available in the literature were assigned fixed values. Fixed values are given in Table 2. The other parameter values were taken from posterior distributions by fitting the model to data (see 2.4 for an outline of methods and summary of statistics of parameters).

Table 1: **Model formulation (human dynamics).**

| **Event** | **Transition** | **Rate** |
|---|---|---|
| Recovery from hospitalisation | $S_{Hi} \to S_{Hi}+1, R_{Hi} \to R_{Hi}-1$ | $\omega_H R_{Hi}$ |
| Natural death of hospitalised | $S_{Hi} \to S_{Hi}+1, R_{Hi} \to R_{Hi}-1$ | $\mu_H R_{Hi}$ |
| Exposure of susceptibles | $S_{Hi} \to S_{Hi}-1, E_{Hi} \to E_{Hi}+1$ | $f_{Hi}\alpha m_{eff} S_{Hi} I_V / N_{Hi}$ |
| Progression to Stage 1 infection | $E_{Hi} \to E_{Hi}-1, I_{1Hi} \to I_{1Hi}+1$ | $\sigma_H E_{Hi}$ |
| Natural death of exposed | $E_{Hi} \to E_{Hi}-1, S_{Hi} \to S_{Hi}+1$ | $\mu_H E_{Hi}$ |
| Progression to Stage 2 infection | $I_{1Hi} \to I_{1Hi}-1, I_{2Hi} \to I_{2Hi}+1$ | $\varphi_H I_{1Hi}$ |
| Natural death of Stage 1 infection | $I_{1Hi} \to I_{1Hi}-1, S_{Hi} \to S_{Hi}+1$ | $\mu_H I_{1Hi}$ |
| Treatment or death from Stage 2 infection | $S_{Hi} \to S_{Hi}+1, I_{2Hi} \to I_{1Hi}-1$ | $\gamma_H I_{2Hi}$ |
| Natural death of Stage 2 infection | $I_{2Hi} \to I_{2Hi}-1, S_{Hi} \to S_{Hi}+1$ | $\mu_H I_{2Hi}$ |

Table 2: **Model parameterisation (fixed parameters).** Notation, a brief description, and the used values for fixed parameters.

| **Notation** | **Description** | **Value** | |
|---|---|---|---|
| $N_H$ | Total human population size in 2015 | Fixed for each health zone | [13] |
| $\mu_H$ | Natural human mortality rate | 5.4795$\times10^{-5}$ days$^{-1}$ | [14] |
| $B_H$ | Total human birth rate | $= \mu_H N_H$ | |
| $\sigma_H$ | Human incubation rate | 0.0833 days$^{-1}$ | [15] |
| $\varphi_H$ | Stage 1 to 2 progression rate | 0.0019 days$^{-1}$ | [16, 17] |
| $\omega_H$ | Recovery rate or waning-immunity rate | 0.006 days$^{-1}$ | [18] |
| Sens | Active screening diagnostic sensitivity | 0.91 | [19] |
| $B_V$ | Tsetse birth rate | 0.0505 days$^{-1}$ | [3] |
| $\xi_V$ | Pupal death rate | 0.037 days $^{-1}$ | |
| $K$ | Pupal carrying capacity | $= 111.09 N_H$ | [3] |
| $\mathbb{P}$(pupating) | Probability of pupating | 0.75 | |
| $\mu_V$ | Tsetse mortality rate | 0.03 days$^{-1}$ | [15] |
| $\sigma_V$ | Tsetse incubation rate | 0.034 days$^{-1}$ | [20, 21] |
| $\alpha$ | Tsetse bite rate | 0.333 days$^{-1}$ | [22] |
| $p_V$ | Probability of tsetse infection per single infective bite | 0.065 | [15] |
| $\varepsilon$ | Reduced non-teneral susceptibility factor | 0.05 | [2] |
| $f_H$ | Proportion of blood-meals on humans | 0.09 | [23] |
| $\mathrm{disp}_{\mathrm{act}}$ | Overdispersion parameter for active detection | 4$\times10^{-4}$ | [1] |
| $\mathrm{disp}_{\mathrm{pass}}$ | Overdispersion parameter for passive detection | 2.8$\times10^{-5}$ | [1] |

[1] Value of $B_V$ is chosen to maintain constant population size without interventions.
[2] Value of $K$ is chosen to reflect the observed bounce back rate.

## 2.4 Summary of previous fitting

The deterministic ODE version of Model W was fitted to health-zone-level data for Bominenge, Budjala, and Mbaya using an adaptive Metropolis-Hastings MCMC algorithm [1]. A summary of the fitted parameter posteriors is given below. In the present analysis, 200 posterior parameter sets were used along with the fitted parameters. To achieve reasonable statistics, we perform 1,000 realisations for each parameter set in the stochastic model.

Table 3: **Model parameterisation (posteriors of fitted parameters).** Notation, a brief description, and representative percentiles of the posterior distributions for fitted parameters.

| Notation | Description | Posterior (median [95% CI]) | | |
|---|---|---|---|---|
| | | **Bominenge** | **Budjala** | **Mbaya** |
| $R_0$ | Basic reproduction number (NGM approach) | 1.04 [1.03, 1.06] | 1.009 [1.006, 1.017] | 1.001 [1.0006 1.0029] |
| $r$ | Relative bites taken on high-risk humans | 1.75 [1.21, 3.15] | 2.47 [1.46, 4.94] | 3.88 [1.74, 8.91] |
| $k_1$ | Proportion of low-risk people | 0.80 [0.69 0.91] | 0.91 [0.77, 0.98] | 0.89 [0.71, 0.97] |
| $\gamma_H^{\text{pre}}$ | Pre-1998 treatment rate from stage 2 (days$^{-1}$) | 2.3$\times10^{-3}$ [1.1, 6.2]$\times10^{-3}$ | 2.9$\times10^{-3}$ [1.3, 7.6]$\times10^{-3}$ | 2.5$\times10^{-3}$ [0.8, 8.1]$\times10^{-3}$ |
| $\eta_H^{\text{post}}$ | Post-1998 treatment rate from stage 1 (days$^{-1}$) | 7$\times10^{-4}$ [4, 10]$\times10^{-4}$ | 5$\times10^{-4}$ [3, 8]$\times10^{-4}$ | 3$\times10^{-4}$ [1, 5]$\times10^{-4}$ |
| $\gamma_H^{\text{post}}$ | Post-1998 treatment rate from stage 2 (days$^{-1}$) | 3.8$\times10^{-3}$ [2.2, 8.7]$\times10^{-3}$ | 4.2$\times10^{-3}$ [2.1, 9.6]$\times10^{-3}$ | 3.4$\times10^{-3}$ [1.2, 10.0]$\times10^{-3}$ |
| Spec | Active screening diagnostic specificity | 0.9998 [0.9997 1.0] | 0.9998 [0.9996, 1.0] | 0.9996 [0.9993, 0.9999] |
| $u$ | Proportion of stage 2 passive cases reported | 0.45 [0.32 0.58] | 0.38 [0.25, 0.5] | 0.36 [0.25, 0.48] |
| $d_{\text{change}}$ | Midpoint year for passive improvement | – | – | – |
| $\eta_{H_{\text{amp}}}$ | Relative improvement in passive stage 1 detection rate | – | – | – |
| $\gamma_{H_{\text{amp}}}$ | Relative improvement in passive stage 2 detection rate | – | – | – |
| $d_{\text{steep}}$ | Speed of improvement in passive detection rate (years$^{-1}$) | – | – | – |

## 2.5 Key updates of the model

This work provides a stochastic description of gHAT dynamics by considering random characteristics of human populations. It allows the population of human compartments to be integer variables and depicts the transitions between them with stochastic events, like Model W in Castaño *et al.* [8]. Whereas the original model presents a mean-field picture of smooth changes of human populations described by continuous variables [1,2]. Our current model accounts for the stochastic nature of different events such as human exposure, recovery, progression, and death. Therefore, individual simulations lead to different infection trajectories, whose averages follow the mean-field deterministic results. The discrete representation of human populations let us define elimination clearly, however in the ODE model an arbitrary threshold is set to define elimination. This stochastic model varies from that in Davis *et al.* [7] by including a Stage 1 passive detection rate and by simulating health zone, rather than village-level population sizes.

# 3 Model S

## 3.1 Description

The Model S used in this study is a stochastic version of a variant of the ordinary differential equations (ODE) model presented in [8], which is based on the initial model published in [24]. The model describes the transmission dynamics of *gambiense* human African trypanosomiasis (gHAT), and consists of a system of coupled ODEs, with compartments for tsetse, animal and human populations. These three different host types are modelled for two different settings corresponding to a low transmission area (e.g. the village, $L$) and a high transmission area (such as river banks or plantations, $H$) that enable accounting for heterogeneity in exposure to tsetse bites. The population size for tsetse, animal or humans in each setting $i$ ($i \in \{L, H\}$) is assumed to be stable by allowing the associated birth terms to compensate deaths in all the compartments. Tsetse and animal populations always stay within their setting (for example, tsetse in low transmission settings always remain in the low transmission setting and animals in high transmission settings always remain in the high transmission setting). Whilst humans in low transmission settings always remain in low transmission setting, humans in the high transmission setting move back and forth between the high and low transmission settings spending a fixed amount of time in each one (to model, for example, the movement of high risk individuals between villages and plantations) — as shown in Figure 2.

Five compartments describe humans in any of the two settings: susceptible ($S_{hi}$); exposed or incubating ($E_{hi}$); infected with the first stage of the disease ($I_{h1i}$); infected with the second stage of the disease, where trypanosomes have reached the cerebro-spinal fluid ($I_{h2i}$); and removed ($T_{hi}$). The total human population in setting $i$ is $N_{hi} = S_{hi} + E_{hi} + I_{h1i} + I_{h2i} + T_{hi}$.

Tsetse populations are divided into susceptible ($S_{vi}$); teneral ($U_{vi}$); exposed ($E_{vi}$); and infected ($I_{vi}$), so that the vector population is $N_{vi} = S_{vi} + U_{vi} + E_{vi} + I_{vi}$. Infected humans in both infected stages can transmit the parasite to tsetse flies. Animals do not contribute to transmission, thus animal populations are modelled as constant parameters, $N_{ai}$, and only form a sink for tsetse bite. A schematic of the model is shown in Figure 2.

The deterministic ODE version of Model S was fitted three times to different health-zone-level data (Bominenge, Bujdala and Mbaya health zones) using an adaptive Metropolis-Hastings Markov chain Monte Carlo (MCMC) algorithm (more information in additional SI document). Stochastic simulations in this analysis used 200 posterior parameter sets sampled from a total of 10,000 samples. To achieve reasonable statistics, we performed 1,000 realisations of the stochastic model for each parameter set.

In this study, the model dynamics is described by random events captured by an adaptive tau-leap algorithm [25], implemented using the 'adaptivetau' R package [26] with the default setting. This adaptive scheme defines the time increment of the simulations in an adaptive manner depending on the state of the system at each time step. The adaptive tau-leaping algorithm dynamically switches between three methods for simulating events: either an explicit or implicit tau-leaping [25], in which the number of events taking place in a time interval $\tau$ is chosen randomly from a Poisson distribution with the mean given by the event rate defining transition, multiplied by time step $\tau$; or an exact method, that is Gillespie's direct method [27], when the leap condition is violated, thus avoiding the issue of negative populations that can happen with tau-leap methods.

## 3.2 Screening

Active screening is modelled via a constant annual detection rate $r_{as}$ that removes infected people only from the low risk setting. As in previous works, we followed [28] to relate a proportion, $d$, of humans effectively screened in a given year, and the annual removal rate, $r_{as}$, as $d = 1 - e^{-r_{as}}$, leading to $r_{as} = -\ln(1-d)$. For the model fitting, screening levels were informed from data, and estimates for each health zone population in 2015 were taken from [13], and projected backwards and forward in time assuming a 3% annual growth rate. For model projections, the mean number of people screened from the last 5 years of available data (2014–2018) was used to define $d$, with an ongoing 3% growth rate in the total population (leading to a continuing decrease in the proportion of the population screened). For projections assuming a 50% coverage for active screening in 2021, we additionally assumed an improved specificity for that year (specificity=1), which has the effect of disabling potential false positives in reported cases.

Passive detection is represented by a continuous stage-specific detection rate, $r1$ and $r2$ for stage 1 and stage 2 respectively, and removes infected people from both low- and high-risk settings. Improvement to passive detection

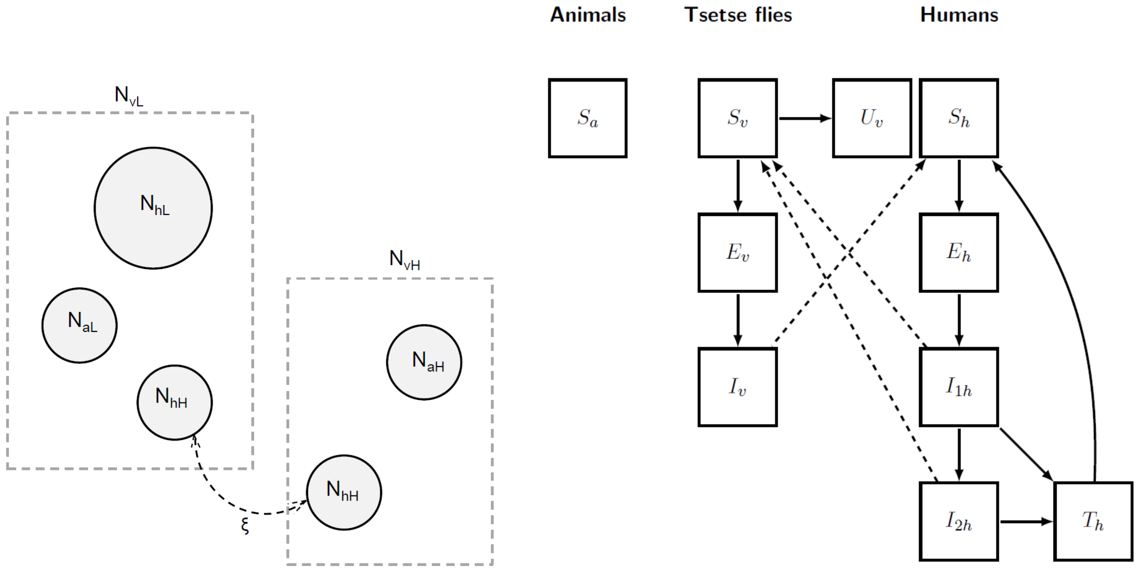


Figure 2: Schematic of Model S. Left: model population structure. Human populations are composed by a stationary population ($N_{hL}$) that remains in low exposure habitats (e.g., a village), and a smaller population ($N_{hH}$) which commute and spend a proportion $\xi$ of their time in a potentially high exposure setting (e.g., a plantation). Each habitat also contains tsetse ($N_{vL}$ and $N_{vH}$) and non-human vertebrate animal populations ($N_{aL}$ and $N_{aH}$). Right: schematic of infection dynamics, subscripts $i \in \{L, H\}$ were removed for easy reading. Compartmental diagram highlights the transmissions between states of infection of the tsetse and human populations, with solid lines indicating transition between compartments, and dashed lines representing transmission rates. Animals cannot transmit infection, thus acting as a sink for tsetse bite. Note that in the low-risk transmission setting, both human populations are exposed to tsetse bites. Figure adapted from [24].

was assumed for the data period. We modelled improvement for the number of years $y > 0$ after 2000 as a logistic function:

$$r1(y) = 365 \times r1_{\text{const}} + \frac{\Delta r_1}{1 + \exp(-\alpha_{\text{pd}}(y - x_0 + 1))},$$

$$r2(y) = 365 \times r1_{\text{const}} \times c2 + \frac{\Delta r_1 + \Delta r_2}{1 + \exp(-\alpha_{\text{pd}}(y - x_0 + 1))},$$

where $r1_{\text{const}}$ and $c_2 * r1_{\text{const}}$ are the constant daily passive detection rates in stage 1 and stage 2 respectively for any time before 2000, and $\Delta r_1$, $\Delta r_2$, $\alpha_{\text{pd}}$ and $x_0$ are parameters defining the profile of the logistic curve. All parameters in the expression above are fitted to the health zone level data. A summary of daily rates at the beginning and end of the data period used for fitting is given in Table 4. For model projections, we assumed passive detection rates $r1$ and $r2$ continue at the highest level from 2000–2016.

Our model assumes that before 2000 only passive detection was ongoing, at constant rates, and that active screening activities started in 2000, the initial year for which there is available data on active screening.

Table 4: **Model S passive detection rates (in days$^{-1}$) over time.**

| | Bominenge ($\times 10^{-3}$) [2.5, 25, 50, 75 and 97.5% CIs] | Budjala ($\times 10^{-3}$) [2.5, 25, 50, 75 and 97.5% CIs] | Mbaya ($\times 10^{-3}$) [2.5, 25, 50, 75 and 97.5% CIs] |
|---|---|---|---|
| Stage 1 rate (2000) | $[1.12, 1.24, 1.31, 1.39, 1.53]$ | $[0.758, 0.855, 0.904, 0.967, 1.09]$ | $[0.377, 0.543, 0.665, 0.797, 1.29]$ |
| Stage 1 rate (2016) | $[1.22, 1.38, 1.48, 1.59, 1.86]$ | $[1.66, 2.09, 2.44, 2.86, 3.79]$ | $[0.63, 1.09, 1.43, 1.86, 2.91]$ |
| Stage 2 rate (2000) | $[1.26, 1.45, 1.59, 1.80, 2.52]$ | $[0.851, 0.978, 1.077, 1.19, 1.46]$ | $[0.521, 0.777, 0.989, 1.25, 2.39]$ |
| Stage 2 rate (2016) | $[1.42, 1.61, 1.79, 2.08, 2.94]$ | $[1.84, 2.44, 2.88, 3.49, 4.80]$ | $[0.884, 1.60, 2.14, 2.97, 5.14]$ |

## 3.3 Parameter values

Descriptions and values of all fixed parameters used in the model are given in Table 5. The remaining parameters were sampled from posterior distributions (200 samples out of 10,000 samples) by fitting the model to health zone case data.

Table 5: **Model parameterisation (fixed parameters).** Notation, a brief description, and the used values of fixed parameters in Model S.

| Notation | Description | Value |
|---|---|---|
| $\alpha$ | Rate at which tsetse become non-teneral (i.e. cannot get infectious) | 73 year$^{-1}$ |
| $A/H_1$ | Density of animals relative to humans in area $L$ | 1.35 |
| $A/H_2$ | Density of animals relative to humans in area $H$ | 1.5 |
| $b$ | Proportion of infective bites leading to infection in humans and animals | 0.433 |
| $c_h$ | Proportion of bites on an infective human that lead to a mature infection in flies | 0.065 |
| $c_{ai}$ | Proportion of bites on an infective animal of type $i$ that lead to a mature infection in flies | 0 |
| $\delta$ | Rate at which treated humans return to the susceptible class | 2.19 year$^{-1}$ |
| $\delta_a$ | Rate of loss of immunity in animal hosts | 0.73 year$^{-1}$ |
| $\eta$ | Rate at which hosts move from the incubating stage | 31.025 year$^{-1}$ |
| $f$ | Inverse of duration of feeding cycle; or biting rate | 121.545 year$^{-1}$ |
| $\gamma$ | Rate of progression to stage 2 in humans | 0.6939 year$^{-1}$ |
| $\gamma_{aL}$ | Rate of progression to the immune class in animal hosts of type $L$ | 1 year$^{-1}$ |
| $\gamma_{aH}$ | Rate of progression to the immune class in animal hosts of type $H$ | 0.6935 year$^{-1}$ |
| $\mu$ | Death rate of humans due to natural causes | 0.01666 year$^{-1}$ |
| $\mu_{ai}$ | Death rate of animal host of type $i$ | 0.584/0.6935 year$^{-1}$ |
| $\mu_\gamma$ | Disease-induced death rate or rate of leaving the recovered state for humans | 1.4484 Year$^{-1}$ |
| $\mu_t$ | Death rate of humans due to treatment | 0 year$^{-1}$ |
| $\mu_v$ | Death rate of tsetse | 10.95 year$^{-1}$ |
| $\nu$ | Inverse of the extrinsic incubation period | 12.41 year$^{-1}$ |
| $\sigma$ | Biting preference for humans | 0.326 |
| $\sigma_{ai}$ | Biting preference for animal in the setting $i$ | 0.8/0.396 |
| $\xi$ | Proportion of time spent i the high risk region by commuters | 0.698 |
| sensitivity | Diagnostics sensitivity (active screening) | 0.91 |

## 3.4 Summary of current fitting

The deterministic ODE version of Model S was fitted to screening and case data for Bominenge, Budjala and Mbaya health zones using an adaptive Metropolis-Hastings MCMC algorithm described in detail in the SI file "Model S — Fitting gHAT model to health zone data". Description of fitted parameters with their posterior median and $95\%$CI are provided in Table 6. Further details in Model S calibration are presented separately in the supplementary information.

Table 6: **Model parameterisation (posteriors of fitted parameters).** Notation, a brief description, and representative percentiles of the posterior distributions for fitted parameters. Here logarithm always refers to the natural logarithm.

| **Notation** | **Description** | **Posterior (median [95% CI])** | | |
|---|---|---|---|---|
| | | **Bominenge** | **Budjala** | **Mbaya** |
| $\kappa$ | Ratio of humans in the high- to low-exposure environment | 1.99 $\times 10^{-2}$ [1.24,3.21]$\times 10^{-2}$ | 4.05 $\times 10^{-3}$ [3.06,5.44]$\times 10^{-3}$ | 1.23 $\times 10^{-3}$ [0.429,3.60]$\times 10^{-3}$ |
| log(VHL) | Log ratio of vectors to humans in low-exposure environment | 1.110 [1.051, 1.179] | 1.18 [1.16, 1.22] | 1.12 [1.02, 1.19] |
| log($c_1$) | Log ratio of the ratio of vectors to humans in the high exposure environment to the same ratio in the low exposure environment | 2.58 $\times 10^{-2}$ [0.13,12.3]$\times 10^{-2}$ | 3.45 $\times 10^{-2}$ [0.02,1.49]$\times 10^{-1}$ | 8.92 $\times 10^{-2}$ [0.01,3.50]$\times 10^{-1}$ |
| logit(spec) | Diagnostic specificity (active screening) in logit scale | 8.129 [7.908, 8.397] | 8.43 [8.04 , 8.99] | 8.62 [7.87 , 9.16] |
| $r1_{\text{const}}$ | Daily passive detection rate for stage 1 (pre-2000) | 4.19$\times 10^{-5}$ [0.21, 17.3]$\times 10^{-5}$ | 1.83$\times 10^{-5}$ [0.10, 8.60]$\times 10^{-5}$ | 7.50$\times 10^{-5}$ [0.35, 41.9]$\times 10^{-5}$ |
| log($c_2$) | Log ratio of passive detection for stage 2 to stage 1 (pre-2000) | 0.165 [0.005, 0.703] | 0.157 [0.005, 0.495] | 0.327 [0.024,1.148] |
| $\Delta r_1$ | Amount passive detection in stage 1 improves | 0.966 [0.831, 1.155] | 1.22 [0.653, 2.34] | 0.644 [0.287, 1.45] |
| $x_0$ | Turning point (years since 1999) for logistic improvement in passive detection | 8.11 [0.44, 16.40] | 9.02 [0.77, 16.26] | 7.14 [0.20, 16.47] |
| $\alpha_{\text{pd}}$ | Steepness in logistic improvement of passive detection | 13.05 $\times 10^{-3}$ [0.24,57.75]$\times 10^{-3}$ | 13.07 $\times 10^{-1}$ [0.769,2.24]$\times 10^{-1}$ | 1.26 $\times 10^{-1}$ [0.078,4.22]$\times 10^{-1}$ |
| $\kappa_{\text{as}}$ | Overdispersion parameter (active screening) | 40.80 [22.30, 68.74] | 62.17 [38.66, 94.18] | 67.93 [44.21, 99.99] |
| $\kappa_{\text{pd}}$ | Overdispersion parameter (passive detection) | 54.39 [34.38, 80.42] | 66.12 [42.37, 97.73] | 69.74 [45.22, 101.91] |

## 3.5 Key updates of the model

The stochastic formulation of gHAT dynamics presented in this work enables capturing the stochastic nature of events involved in transmission dynamics while producing integer outputs (e.g. number of cases and new infections here). This approach permits a clear definition of elimination of transmission and subsequent forecasting of elimination timelines, and it has already been presented in [8].

The underlying deterministic ODE model used for model fitting is built in previous published work [8, 9], with the following main modifications to the way detection and treatment are modelled:

**Active screeening**. We included imperfect sensitivity and specificity in the diagnostic process. While sensitivity is set to 0.91 ( [9], specificity is fitted for each health zone).

**Passive detection**. As in [9], we assume improvement to passive detection. In this implementation, a logistic function is used to define this improvement, with logistic-related parameters fitted to each health zone.

# 4 Model comparison

A comparison of the structures of the two models is shown in Table 4.

Table 7: **Model comparison**. The differences between the modelling assumptions of Model W and Model S.

| | **Model W** | **Model S** |
|---|---|---|
| **Risk structure in human populations** | Yes — high-/ low-risk structure. High-risk not participating in active screening and higher relative risk of tsetse bites. | Yes — high-/ low-risk structure. High-risk not participating in active screening and higher relative risk of tsetse bites. Human mobility of high risk individuals between regions of low and high tsetse exposure. |
| **Model fitting** | Using fitting from deterministic model to 2000–2016 data. Adaptive MCMC method is described in Crump *et al.* [1]. | Using fitting from deterministic model to 2000–2016 data . Adaptive MCMC method and output are found in Aliee *et al.* [29]. |
| **Stochastic model for simulations** | Tau-leap method (one day time step). Tsetse with ODE equations. | Adaptive tau-leap method applied to the whole dynamical system. |
| **Improvements in passive surveillance** | Yes — in 1998 (following the introduction of the CATT test). No change assumed afterwards. | Yes — Constant rates before 2000, improvement assumed to start afterwards. |
| **Active screening** | Occurs once at the beginning of the year. | Continuous rate over each year. |
| **Imperfect specificity** | Fitted for each health zone independently and assumed to be 100% after 2018 given few reported cases (less than 0.5 reported cases above the expected number of false positives per 10,000 people). | Fitted for each health zone independently. |
| **Animal or asymptomatic reservoir** | None assumed. | None assumed. |

# 5 Modelling results

We present the results from Figure 1 of the main manuscript — case reporting and inferred infection dynamics by the two models in three health zones for Sud-Ubangi province, DRC — with the mean values shown instead of the median (Figure 3).

For Figure 2 in the main manuscript, we calculate the probability of EOT using the following definition: EOT is achieved for model simulations in the first of ten consecutive years with no new infections. Thus, EOT represents the last transmission event. We use this 10-year criterion as we note that cases can still be identified long after the last transmission event, particularly as people have been reported as being infected with gHAT for long time periods (in one case over 29 years) [30]. In addition, if the disease is locally eliminated in the human population, the short life span of tsetse means that the infection will be unlikely to persist without human infection [7].

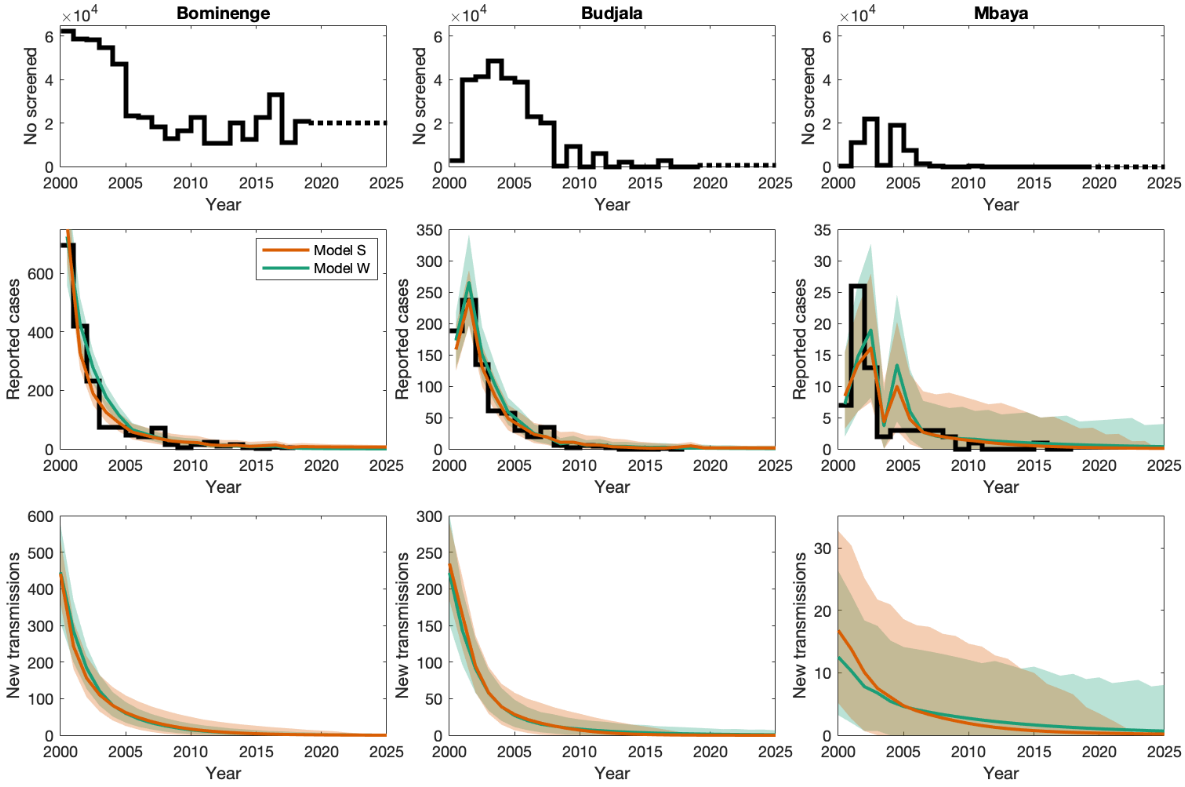


Figure 3: **Case reporting and inferred infection dynamics by the two models in three health zones for Sud-Ubangi province, DRC.** The first row shows the number of people screened in each year in each health zone, with dashed lines representing our assumed future active screening coverage. The second row shows the total reported case data as a black solid line and the model fits as a coloured lines (mean) and shaded area (95% credible and prediction intervals), the last row shows our estimated number of new infections in humans (transmission) over time. Model S is orange and Model W is green.

# 6 NTD-PRIME criteria

Table 8: **PRIME-NTD criteria fulfillment**. How this study has satisfied the five principles of the Neglected Tropical Diseases Modelling Consortium.

| The principle and what has been done to satisfy this principle? | Where in the manuscript is this described? |
|---|---|
| **1. Stakeholder engagement** | |
| The study was led by modellers with guidance from E Mwamba Miaka of the national sleeping sickness control programme in DRC (PNLTHA-DRC). PNLTHA-DRC have provided detailed knowledge of the data and information as to how the programme has changed over time. The initial motivation for the manuscript was determined after discussions with the World Health Organization. | Authorship list. |
| **2. Complete model documentation** | |
| The deterministic model code used for fitting Model W is available on Open-ScienceFramework (OSF) at https://osf.io/ck3tr/. Model code for both models is provided in the supplementary material, where both models are also fully described. | OSF and Supplementary material. |
| **3. Complete description of data used** | |
| Our models are fitted to screening and case data from the WHO HAT Atlas for the selected health zones. The fitting process for Model W is described in detail in [1] and in the supplementary information to this manuscript for Model S. | Methods section of main manuscript, supplementary material and [1]. |
| **4. Communicating uncertainty** | |
| Structural uncertainty: Model selection for Model W was completed in Rock *et al.*, where plausible models were compared.<br>Model parameter uncertainty: Both models were fitted using MCMC methodology with data specific to the health zones considered. 200 parameter sets from the generated posteriors were used to compute the infection dynamics in this study.<br>Stochastic uncertainty: Both models derive expected infection dynamics from 200,000 stochastic realisations. This is a sufficiently large number of simulations to ensure small confidence intervals in the median value of the dynamics. | Methods and supplementary material. |
| **5. Testable model outcomes** | |
| We determine the probability that EOT has been met in three health zones of DRC (Mbaya, Budjala and Bominenge). Continued surveillance in these regions would indicate how robust our predictions are — for example whether the case reporting in subsequent data falls within our case prediction intervals. | Results section of main manuscript. |

# References


[1] RE Crump, CI Huang, E Knock, Spencer SEF, P Brown, EM Miaka, S Chancy, MJ Keeling, and KS Rock. Quantifying epidemiological drivers of gambiense human african trypanosomiasis across the democratic republic of congo. *medRxiv https://doi.org/10.1101/2020.06.23.20138065*, 2020.

[2] Kat S Rock, Steve J Torr, Crispin Lumbala, and Matt J Keeling. Quantitative evaluation of the strategy to eliminate human african trypanosomiasis in the democratic republic of congo. *Parasites & vectors*, 8(1):532, 2015.

[3] Kat S Rock, Steve J Torr, Crispin Lumbala, and Matt J Keeling. Predicting the impact of intervention strategies for sleeping sickness in two high-endemicity health zones of the democratic republic of congo. *PLoS neglected tropical diseases*, 11(1):e0005162, 2017.

[4] K.S. Rock, A. Pandey, M.L. Ndeffo-Mbah, K.E. Atkins, C. Lumbala, A. Galvani, and M.J. Keeling. Data-driven models to predict the elimination of sleeping sickness in former Equateur province of DRC. *Epidemics*, 18:101–112, 2017.

[5] Mahamat Hissene Mahamat, Mallaye Peka, Jean-Baptiste Rayaisse, Kat S Rock, Mahamat Abdelrahim Toko, Justin Darnas, Guihini Mollo Brahim, Ali Bachar Alkatib, Wilfrid Yoni, Inaki Tirados, et al. Adding tsetse control to medical activities contributes to decreasing transmission of sleeping sickness in the mandoul focus (chad). *PLoS neglected tropical diseases*, 11(7):e0005792, 2017.

[6] Kat S Rock, Martial L Ndeffo-Mbah, Soledad Castaño, Cody Palmer, Abhishek Pandey, Katherine E Atkins, Joseph M Ndung'u, T Déirdre Hollingsworth, Alison Galvani, Caitlin Bever, et al. Assessing strategies against gambiense sleeping sickness through mathematical modeling. *Clinical infectious diseases*, 66(suppl_4):S286–S292, 2018.

[7] Christopher N Davis, Kat S Rock, Erick Mwamba Miaka, and Matt J Keeling. Village-scale persistence and elimination of gambiense human african trypanosomiasis. *PLoS neglected tropical diseases*, 13(10):e0007838, 2019.

[8] M Soledad Castaño, Maryam Aliee, Erick Mwamba Miaka, Matt J Keeling, Nakul Chitnis, and Kat S Rock. Screening strategies for a sustainable endpoint for gambiense sleeping sickness. *The Journal of Infectious Diseases*, 221(Supplement_5):S539–S545, 2019.

[9] María Soledad Castaño, Martial L Ndeffo-Mbah, Kat S Rock, Cody Palmer, Edward Knock, Erick Mwamba Miaka, Joseph M Ndung'u, Steve Torr, Paul Verlé, Simon EF Spencer, et al. Assessing the impact of aggregating disease stage data in model predictions of human african trypanosomiasis transmission and control activities in bandundu province (drc). *PLoS neglected tropical diseases*, 14(1):e0007976, 2020.

[10] Ching-I Huang, Ronald E Crump, Paul Brown, Simon EF Spencer, Erick Mwamba Miaka, Chansy Shampa, Matt J Keeling, and Kat S Rock. Shrinking the ghat map: identifying target regions for enhanced control of gambiense human african trypanosomiasis in the democratic republic of congo. *medRxiv*, 2020.

[11] Christopher N Davis, Kat S Rock, Marina Antillon, Erick Mwamba Miaka, and Matt J Keeling. Cost-effectiveness modelling to optimise active screening strategy for gambiense human african trypanosomiasis in the democratic republic of congo. *medRxiv*, 2020.

[12] Maryam Aliee, Kat S Rock, and Matt J Keeling. Estimating the time to extinction of infectious diseases in mean-field approaches. *medRxiv*, 2020.

[13] OCHA Office for the Coordination of Humanitarian Affairs. Journees nationales de vaccination (jnv) activities de vaccination supplementaire, rdc. (accessed May 2016).

[14] The World Bank. Data: Democratic republic of congo. (accessed 2015).

[15] D J Rogers. A general model for the African trypanosomiases. *Parasitology*, 97:193–212, 1988.

[16] F Checchi, J A N Filipe, M P Barrett, and D Chandramohan. The natural progression of gambiense sleeping sickness: what is the evidence? *PLOS Neglected Tropical Diseases*, 2(12):e303, December 2008.

[17] F Checchi, S Funk, D Chandramohan, D T Haydon, and F Chappuis. Updated estimate of the duration of the meningo-encephalitic stage in gambiense human African trypanosomiasis. *BMC Research Notes*, 8(1):292, July 2015.

[18] A Mpanya, D Hendrickx, M Vuna, A Kanyinda, C Lumbala, V Tshilombo, P Mitashi, O Luboya, V Kande, M Boelaert, P Lefèvre, and P Lutumba. Should i get screened for sleeping sickness? a qualitative study in kasai province, democratic republic of congo. *PLOS Neglected Tropical Diseases*, 6(1):e1467, January 2012.

[19] F Checchi, F Chappuis, Unni Karunakara, Gerardo Priotto, and D Chandramohan. Accuracy of five algorithms to diagnose gambiense human african trypanosomiasis. *PLOS Neglected Tropical Diseases*, 5(7):e1233, July 2011.

[20] S Davis, S Aksoy, and A P Galvani. A global sensitivity analysis for African sleeping sickness. *Parasitology*, 138(04):516–526, November 2010.

[21] S Ravel, P Grebaut, D Cuisance, and G Cuny. Monitoring the developmental status of Trypanosoma brucei gambiense in the tsetse fly by means of PCR analysis of anal and saliva drops. *Acta Tropica*, 88(2):161–165, October 2003.

[22] World Health Organization. Control and surveillance of human african trypanosomiasis: report of a who expert committee. Technical report, 2013. (accessed April 20, 2020).

[23] P-H Clausen, I Adeyemi, B Bauer, M Breloeer, F Salchow, and C Staak. Host preferences of tsetse (Diptera: Glossinidae) based on bloodmeal identifications. *Medical and Veterinary Entomology*, 12(2):169–180, March 1998.

[24] Chris M Stone and Nakul Chitnis. Implications of heterogeneous biting exposure and animal hosts on trypanosomiasis brucei gambiense transmission and control. *PLoS computational biology*, 11(10):e1004514, 2015.

[25] Yang Cao, Daniel T Gillespie, and Linda R Petzold. Adaptive explicit-implicit tau-leaping method with automatic tau selection. *The Journal of chemical physics*, 126(22):224101, 2007.

[26] Philip Johnson. adaptivetau: Tau-leaping stochastic simulation. *R package version 0.902*, 2011.

[27] Daniel T Gillespie. A general method for numerically simulating the stochastic time evolution of coupled chemical reactions. *Journal of computational physics*, 22(4):403–434, 1976.

[28] Marc Artzrouni and Jean-Paul Gouteux. A compartmental model of sleeping sickness in central Africa. *Journal of Biological Systems*, 4(04):459–477, 1996.

[29] Maryam Aliee, Soledad Castano, Christopher N Davis, Swati Patel, Erick Mwamba Miaka, Simon EF Spencer, Matt J Keeling, Nakul Chitnis, and Kat S Rock. Predicting the impact of COVID-19 interruptions on transmission of gambiense human african trypanosomiasis in two health zones of the Democratic Republic of Congo. *medRxiv*, 2020.

[30] Darshan Sudarshi, Sarah Lawrence, William Owen Pickrell, Vinay Eligar, Richard Walters, Shumonta Quaderi, Alice Walker, Paul Capewell, Caroline Clucas, Angela Vincent, et al. Human african trypanosomiasis presenting at least 29 years after infection—what can this teach us about the pathogenesis and control of this neglected tropical disease? *PLoS Negl Trop Dis*, 8(12):e3349, 2014.
