# Supplementary material for: Modelling to Quantify the Likelihood that Local Elimination of Transmission has Occurred Using Routine Gambiense Human African Trypanosomiasis Surveillance Data
Source: Clin Infect Dis. 2021 Jun 14;72(Suppl 3):S146–51. doi: 10.1093/cid/ciab190 (PMC8201550; doi:10.1093/cid/ciab190)
Supplement: ciab190_suppl_Supplementary-Data [file ciab190_suppl_supplementary-data.pdf]

Supplementary information
Model S - Fitting gHAT model to health zone data

This is a complimentary document to the main Supplementary Information material attached to the manuscript *Modelling to quantify the likelihood that local elimination of transmission has occurred using routine gambiense human African trypanosomiasis surveillance data*.
This document presents additional information on Model S calibration to three health zones of the former Equateur province in the Democratic Republic of Congo, namely Bominenge, Budjala and Mbaya health zones.

1 Fitting procedure

The model was fit three times to the different health zone data sets. In each case, eleven parameters were fitted using annual case data from the period 2000–2016. The model was first run to reach equilibrium prevalence of infection assuming only constant passive screening before 2000, after which the fitting starts. The data classified reported cases into those detected through active screening and from passive detection; and from 2015 onwards, further provided information on disease staging. Fitting was performed via an adaptive Metropolis-Hastings Markov chain Monte Carlo (MCMC) approach, and used the following log-likelihood function:

$$
\begin{aligned}
LL(\theta|x) = & \log(P(x|\theta)) \\
\propto & \sum_{i=2000}^{2016 \text{ or } 2018} \Bigg(\log\left[\mathrm{NegBin}\left(A_{d1}(i)+A_{d2}(i), A_{m1}(i)+A_{m2}(i), \kappa_{\mathrm{AS}}\right)\right] \\
& + \log\left[\mathrm{NegBin}\left(P_{d1}(i)+P_{d2}(i), P_{m1}(i)+P_{m2}(i), \kappa_{\mathrm{PD}}\right)\right] \Bigg) \\
& + \sum_{i=2015}^{2016 \text{ or } 2018} \Bigg(\log\left[\mathrm{Bin}\left(P_{d1}(i), P_{d1}(i)+P_{d2}(i), \frac{P_{m1}(i)}{P_{m1}(i)+P_{m2}(i)}\right)\right] \\
& + \log\left[\mathrm{Bin}\left(A_{d1}(i), A_{d1}(i)+A_{d2}(i), \frac{A_{m1}(i)}{A_{m1}(i)+A_{m2}(i)}\right)\right] \Bigg).
\end{aligned}
$$

The first two terms in the inner sum represent the total number of active and passive cases in the data, respectively, modeled as a negative binomial with mean equal to the number of cases from the differential equation model. The second two terms in the inner sum represent the proportion of cases in stage 1, modeled as a binomial in which the probability of stage 1 is the proportion from the differential equation model (and the number of trials is from the data of total number of cases). Since we only have staged data for after 2015, those terms only contribute to the log likelihood in those years. Nomenclature for the expression above is: A_{d1}: stage 1 reported cases (active screening); A_{d2}: stage 2 reported cases (active screening); P_{d1}: stage 1 reported cases (passive detection); P_{d2}: stage 2 reported cases (passive detection); A_{m1}: stage 1 reported cases from the model (active screening);

A_{m2}: stage 2 reported cases from the model (active screening); P_{m1}: stage 1 reported cases from the model (passive surveillance); P_{m2}: stage 2 reported cases from the model (passive surveillance).

To sample from the posterior distribution, determined by the likelihood function and the prior distributions, we used an adaptive Metropolis-Hastings MCMC algorithm (unpublished, Spencer). For the sampling, we ran two independent chains of the algorithm to corroborate convergence. We used a burn-in period of 2000 steps and then ran the chain for 20,000 steps, which was thinned to every other sample. For the proposal distribution, we used a multivariate Normal distribution (truncated with the bounds given in Table 1), with a covariance matrix that adapts to predict the shape and scale of the posterior distribution as the algorithm proceeds. The adaptation improves the efficiency of proposing new samples so that there are neither excessive rejections nor acceptances in the algorithm. Finally, to improve mixing further, we used the two covariance matrices from this first set of runs in a second round of two independent chains with the same burn-in and sampling strategy. With this second set, we visually checked that there was good mixing. The parameters used in the forward projections are based on the first chain of this second set of runs.

1.1 Fixed parameters, priors and posterior distributions

Descriptions and values of all fixed parameters are given in Table 5 of the main Supplementary Information file. Descriptions, prior distributions (Table 1) and summary of posterior distributions (Table 2) for all fitted parameters are presented below.

Parameter	Unit	Prior Distribution	Bounds
κ	-	Unif$[0, 1]$	$[0, 1]^*$
log(VHL)	-	N$(1.1, 0.05)$	$[0, \log(100)]$
log(c_1)	-	Gamma$(1, 1)$	$[0, \log(50)]$
logit(spec)	-	Unif$[\text{logit}(0.999), \text{logit}(0.9999)]$	same
$r1_{\text{const}}$	day^{-1}	Unif$[0, 0.001]$	same
log(c_2)	-	Gamma$(1, 1)$	$[0, \log(50)]$
Δr_1	year^{-1}	Unif$[0, 5]$	same
Δr_2	year^{-1}	Unif$[0, 2.5]$	same
x_0	-	Unif$[0, 17]$	same
α_{pd}	-	Unif$[0, 0.5]$	same
κ_{as}	-	Gamma$(23.5, 3)$	same
κ_{pd}	-	Gamma$(23.5, 3)$	same

Table 1: Priors for fitted parameters for Model S. Non-uniform priors were additionally truncated with values given in brackets. Gamma priors are written with arguments of shape and scale. *: Upper bound for kappa depends on population size and screened people via the rate expression used for active screeening; it was set to 0.64 for Bominenge, to 0.67 for Budjala, and to 1 for Mbaya health zone.

Table 2: **Model parameterisation (posteriors of fitted parameters).** Notation, a brief description, and representative percentiles of the posterior distributions for fitted parameters. Here logarithm always refers to the natural logarithm.

Notation	Description	Posterior (median [95% CI])		
		**Bominenge**	**Budjala**	**Mbaya**
κ	Ratio of humans in the high- to low-exposure environment	1.99 $\times 10^{-2}$ [1.24,3.21]$\times 10^{-2}$	4.05 $\times 10^{-3}$ [3.06,5.44]$\times 10^{-3}$	1.23 $\times 10^{-3}$ [0.429,3.60]$\times 10^{-3}$
log(VHL)	Log ratio of vectors to humans in low-exposure environment	1.110 [1.051, 1.179]	1.18 [1.16, 1.22]	1.12 [1.02, 1.19]
log(c_1)	Log ratio of the ratio of vectors to humans in the high exposure environment to the same ratio in the low exposure environment	2.58 $\times 10^{-2}$ [0.13,12.3]$\times 10^{-2}$	3.45 $\times 10^{-2}$ [0.02,1.49]$\times 10^{-1}$	8.92 $\times 10^{-2}$ [0.01,3.50]$\times 10^{-1}$
logit(spec)	Diagnostic specificity (active screening)	8.129 [7.908, 8.397]	8.43 [8.04 , 8.99]	8.62 [7.87 , 9.16]
$r1_{\text{const}}$	Daily passive detection rate for stage 1 (pre-2000)	4.19$\times 10^{-5}$ [0.21, 17.3]$\times 10^{-5}$	1.83$\times 10^{-5}$ [0.10, 8.60]$\times 10^{-5}$	7.50$\times 10^{-5}$ [0.35, 41.9]$\times 10^{-5}$
log(c_2)	Log ratio of passive detection for stage 2 to stage 1 (pre-2000)	0.165 [0.005, 0.703]	0.157 [0.005, 0.495]	0.327 [0.024,1.148]
Δr_1	Amount passive detection in stage 1 improves	0.966 [0.831, 1.155]	1.22 [0.653, 2.34]	0.644 [0.287, 1.45]
x_0	Turning point (years since 1999) for logistic improvement in passive detection	8.11 [0.44, 16.40]	9.02 [0.77, 16.26]	7.14 [0.20, 16.47]
α_{pd}	Steepness in logistic improvement of passive detection	13.05 $\times 10^{-3}$ [0.24,57.75]$\times 10^{-3}$	13.07 $\times 10^{-1}$ [0.769,2.24]$\times 10^{-1}$	1.26 $\times 10^{-1}$ [0.078,4.22]$\times 10^{-1}$
κ_{as}	Overdispersion parameter (active screening)	40.80 [22.30, 68.74]	62.17 [38.66, 94.18]	67.93 [44.21, 99.99]
κ_{pd}	Overdispersion parameter (passive detection)	54.39 [34.38, 80.42]	66.12 [42.37, 97.73]	69.74 [45.22, 101.91]

1.2 MCMC outputs

The MCMC outputs shown in this section correspond to 10,000 post burn-in samples.

1.2.1 Bominenge

Model fit to reported case data (Fig 1), MCMC posterior densities (Fig 2) and traces (Fig 3) for Bominenge health zone.

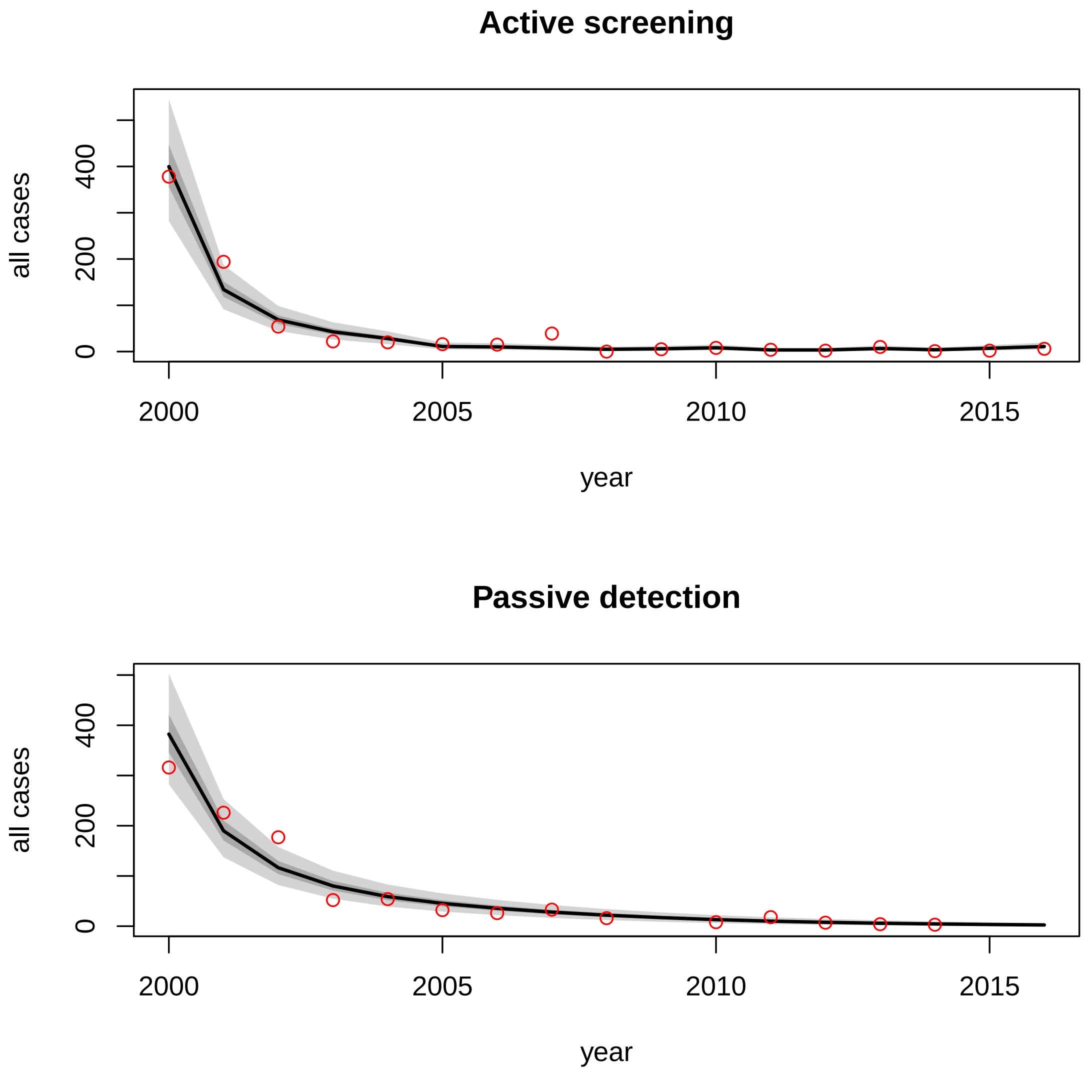

Figure 1: Model fit to reported case data for Bominenge health zone. Median (continuous line), 95%CI (dashed area) and 50%CI (darker shared area) are contrasted to reported case data (red dots).

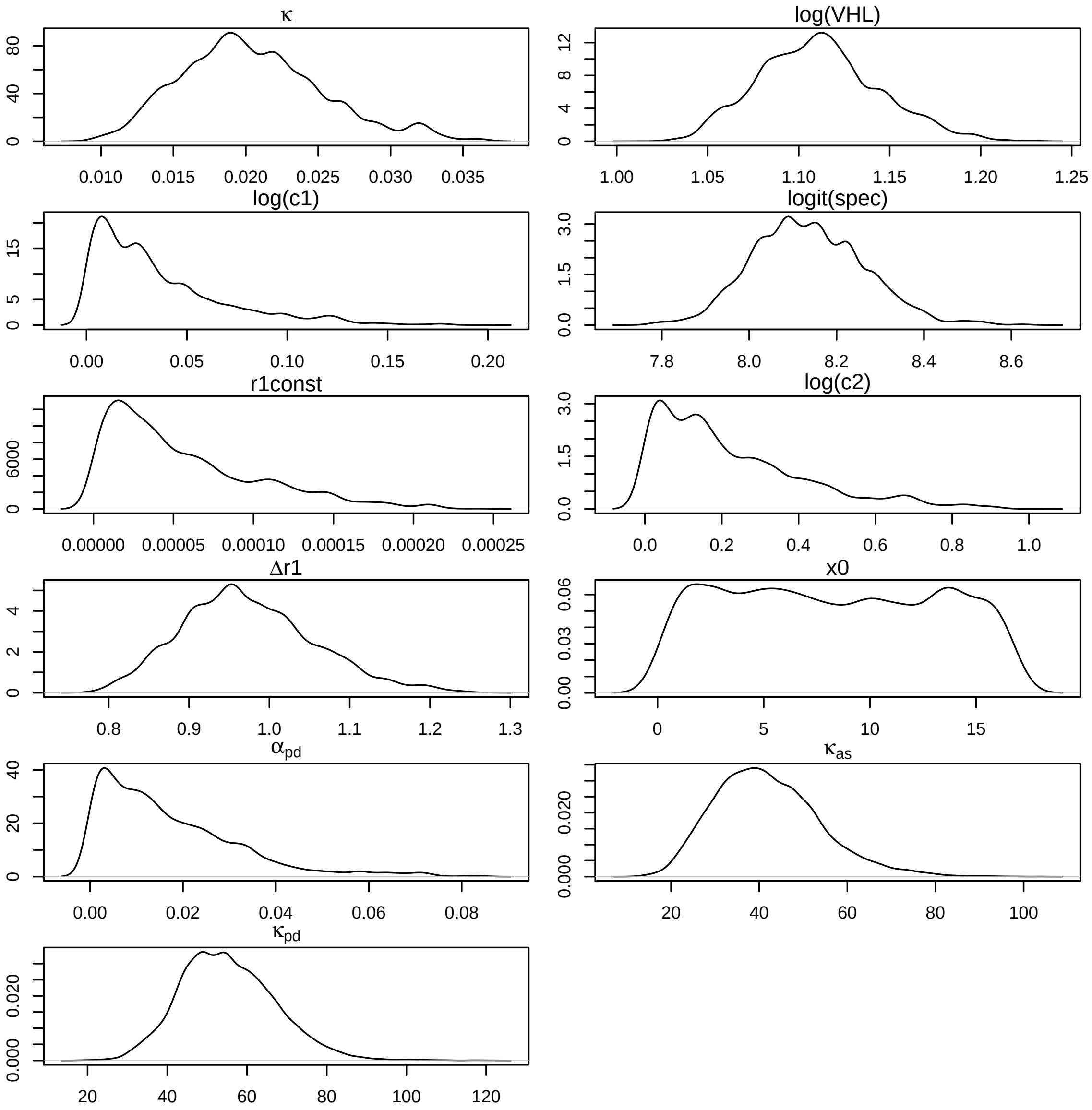

Figure 2: Posterior density of fitted parameters for Bominenge health zone.

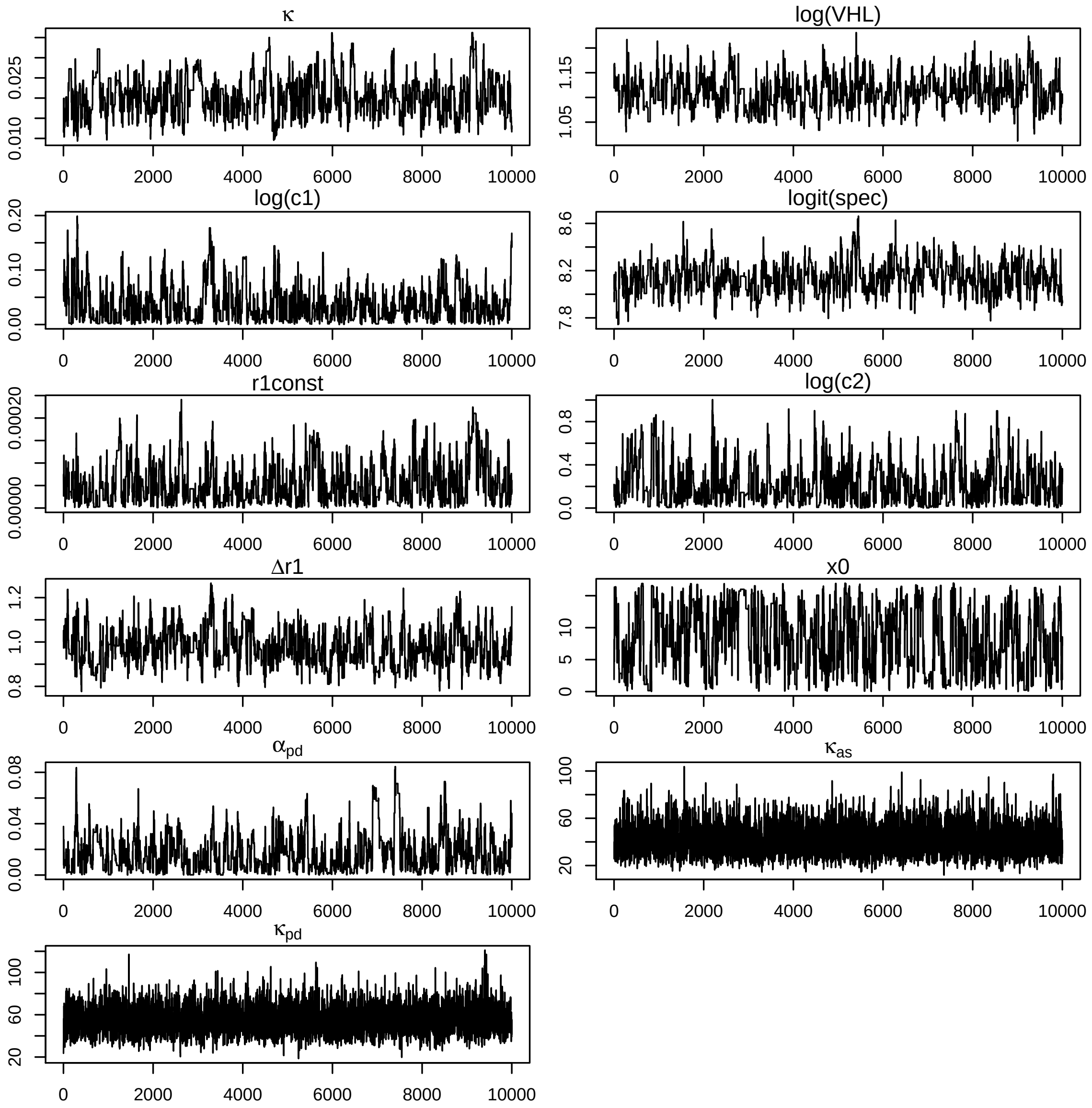

Figure 3: Traces of fitted parameters for Bominenge health zone.

1.2.2 Budjala

Model fit to reported case data (Fig 4), MCMC posterior densities (Fig 5) and traces (Fig 6) for Budjala health zone.

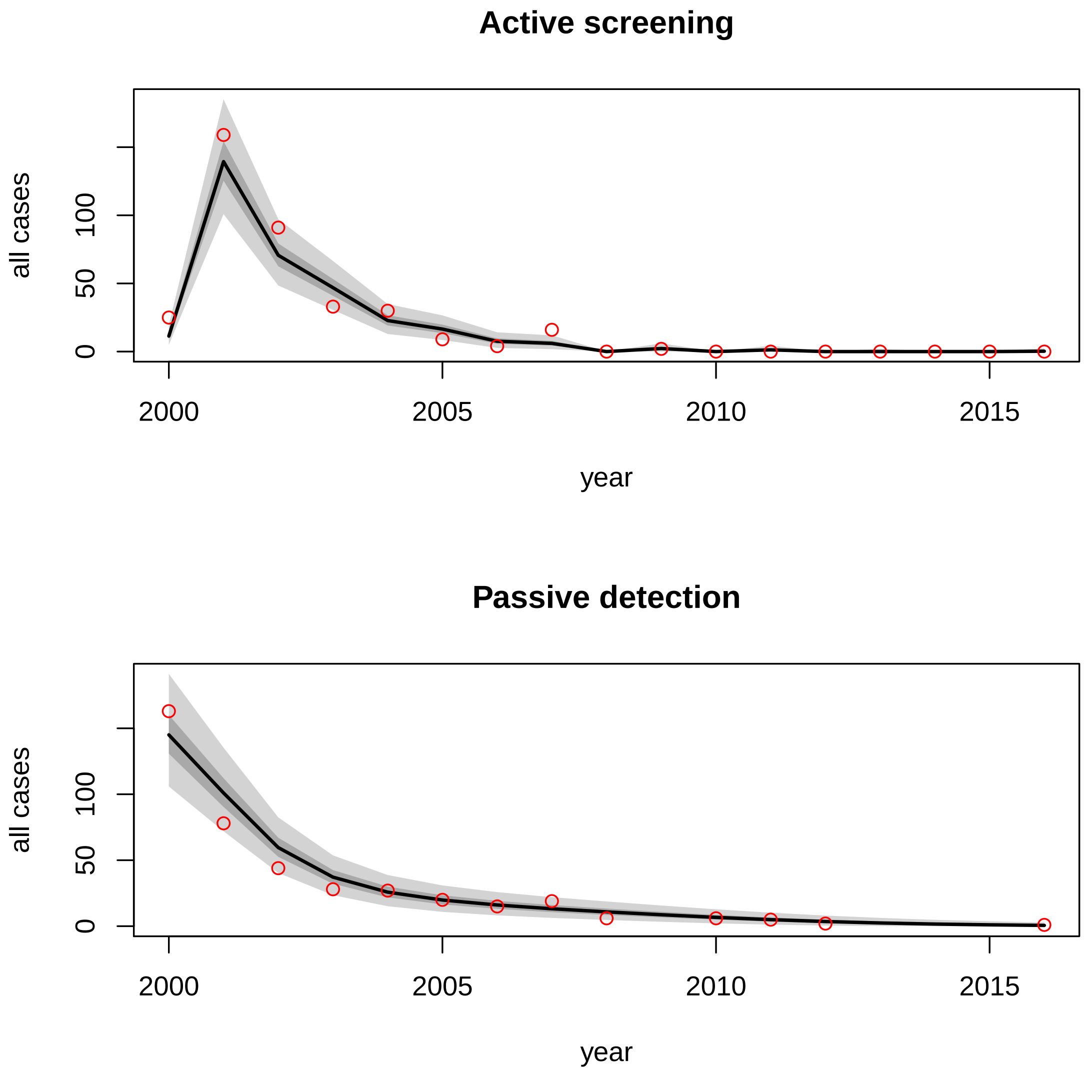

Figure 4: Model fit to reported case data for Budjala health zone. Median (continuous line), 95%CI (dashed area) and 50%CI (darker shared area) are contrasted to reported case data (red dots).

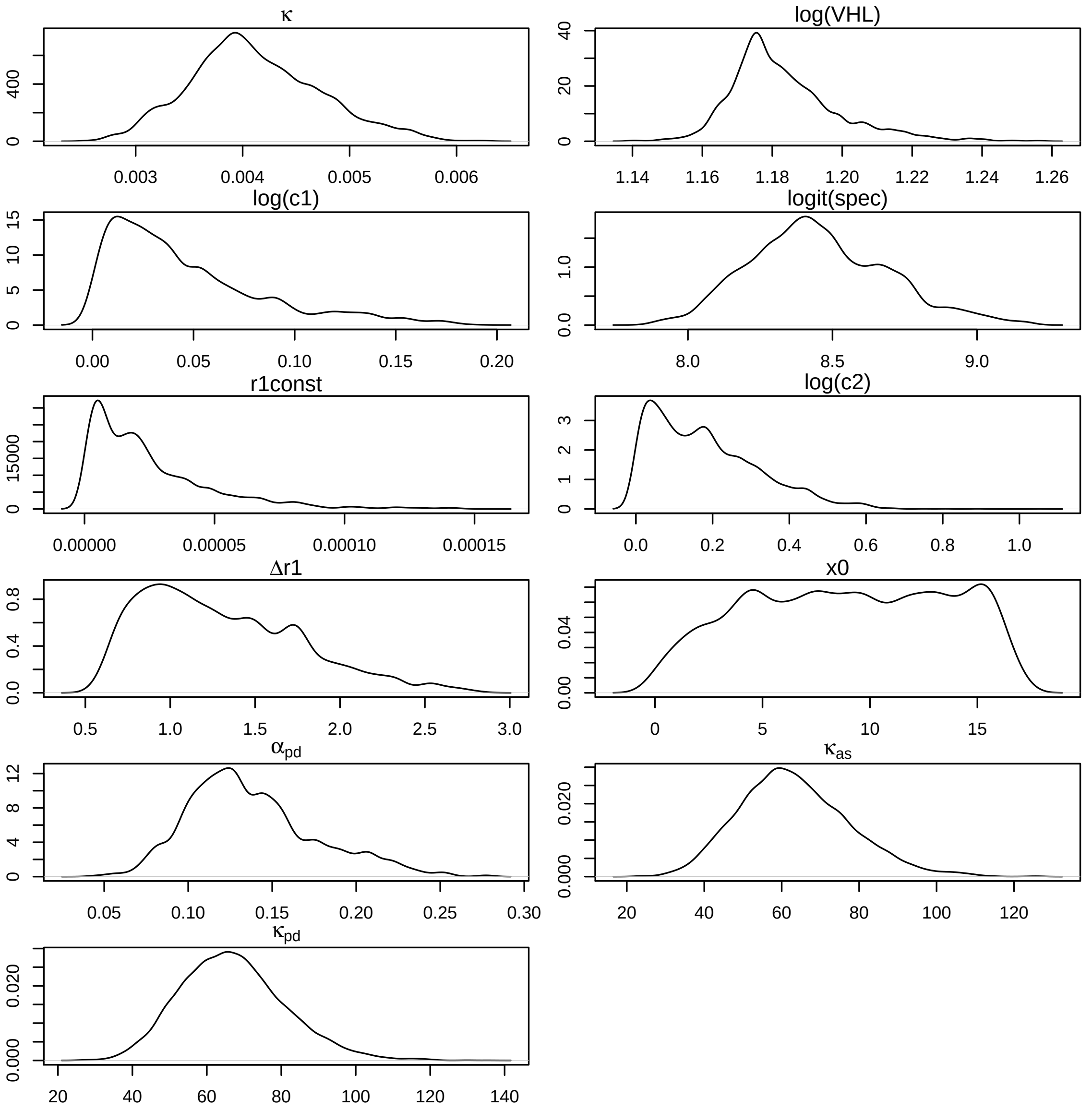

Figure 5: Posterior density of fitted parameters for Budjala health zone.

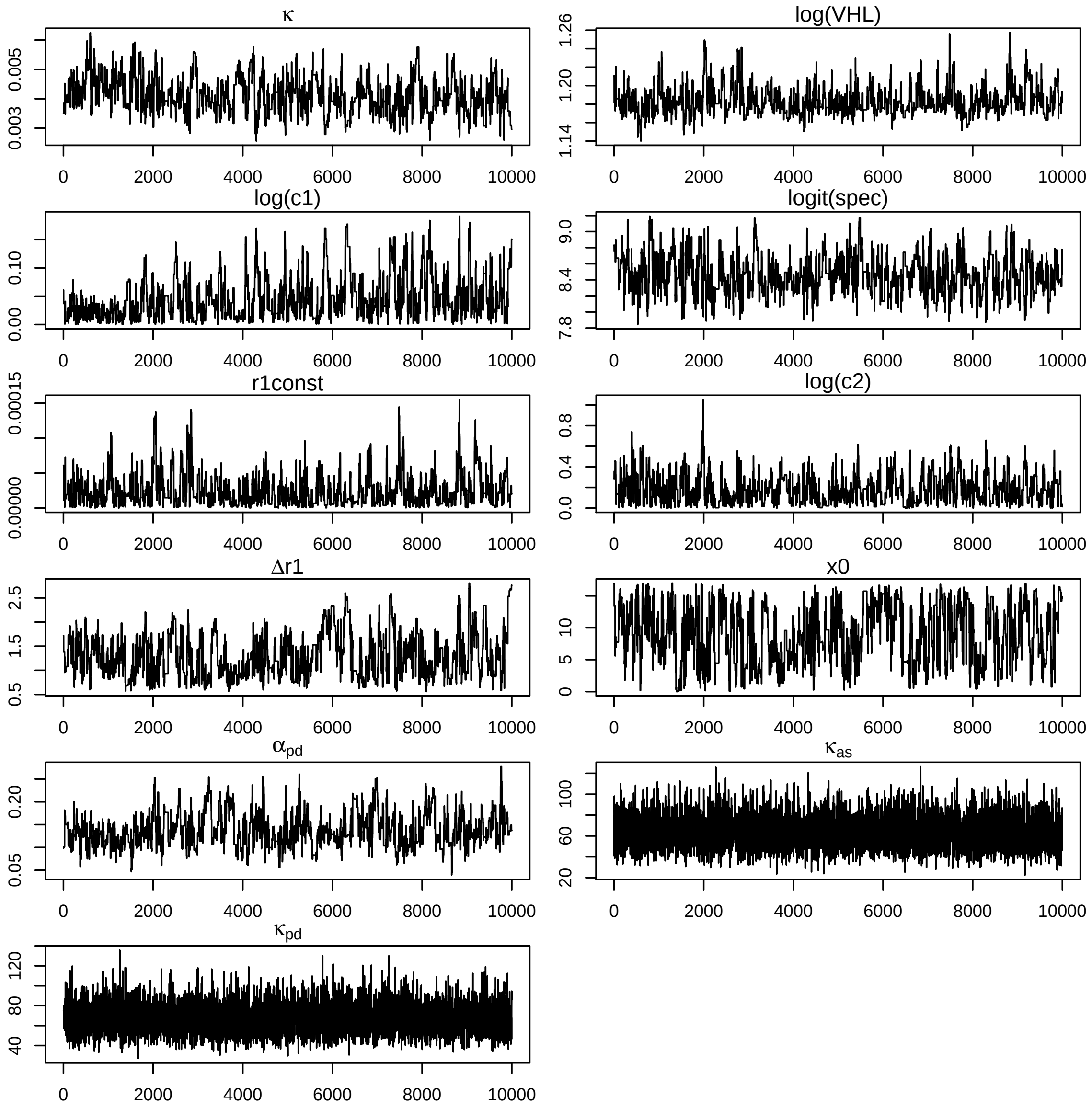

Figure 6: Traces of fitted parameters for Budjala health zone.

1.2.3 Mbaya

Model fit to reported case data (Fig 7), MCMC posterior densities (Fig 8) and traces (Fig 9) for Mbaya health zone.

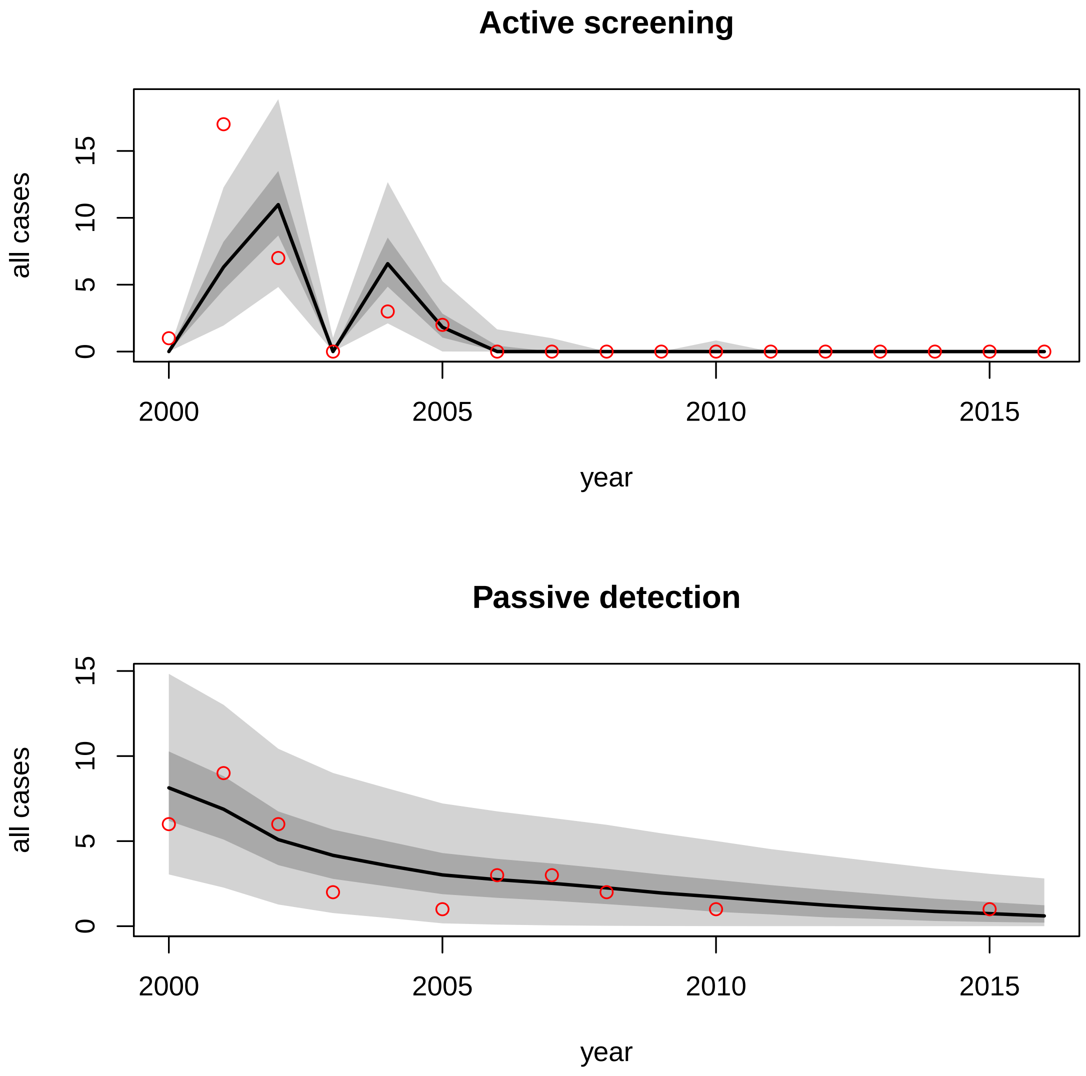

Figure 7: Model fit to reported case data for Mbaya health zone. Median (continuous line), 95%CI (dashed area) and 50%CI (darker shared area) are contrasted to reported case data (red dots).

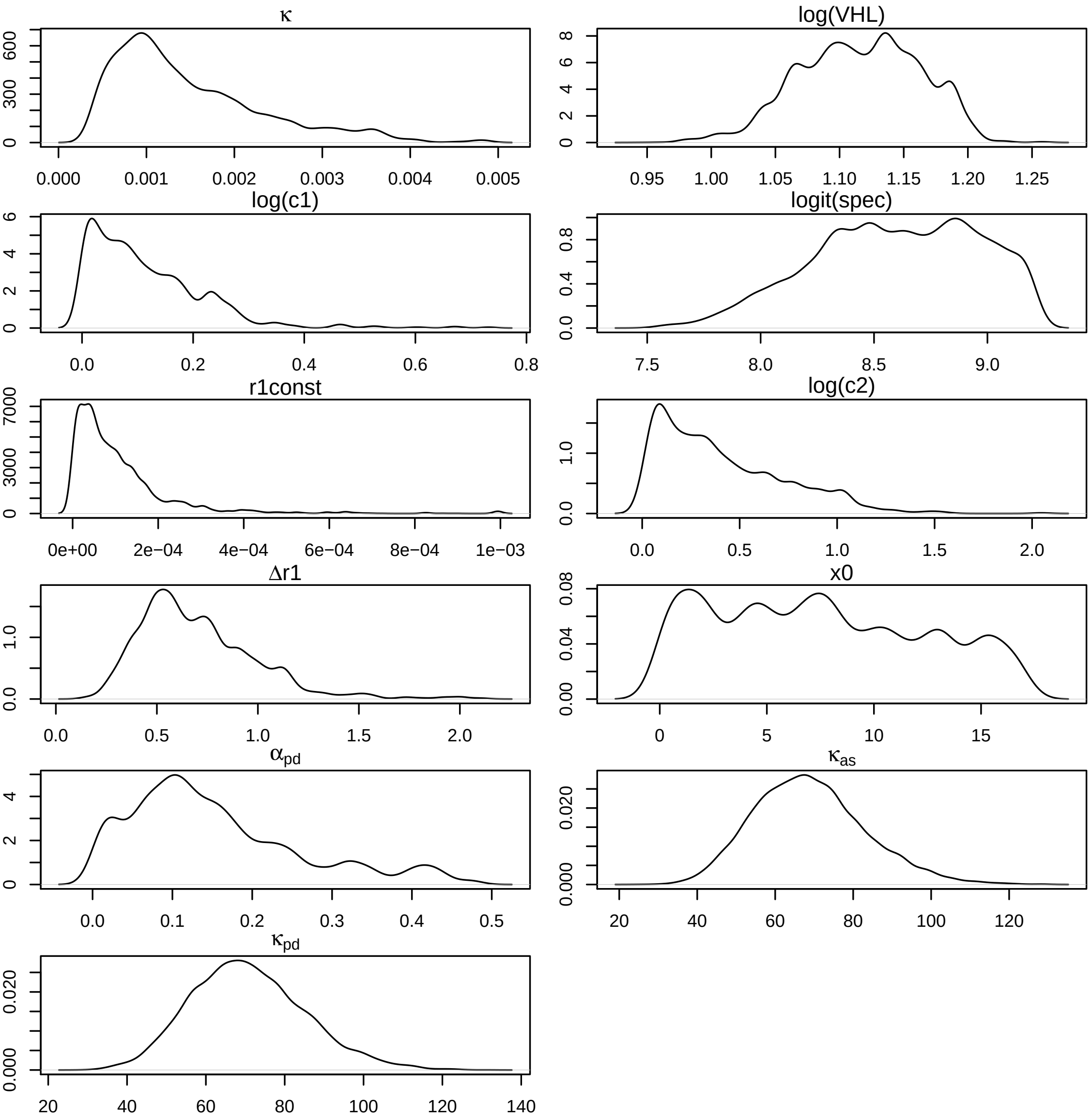

Figure 8: Posterior density of fitted parameters for Mbaya health zone.

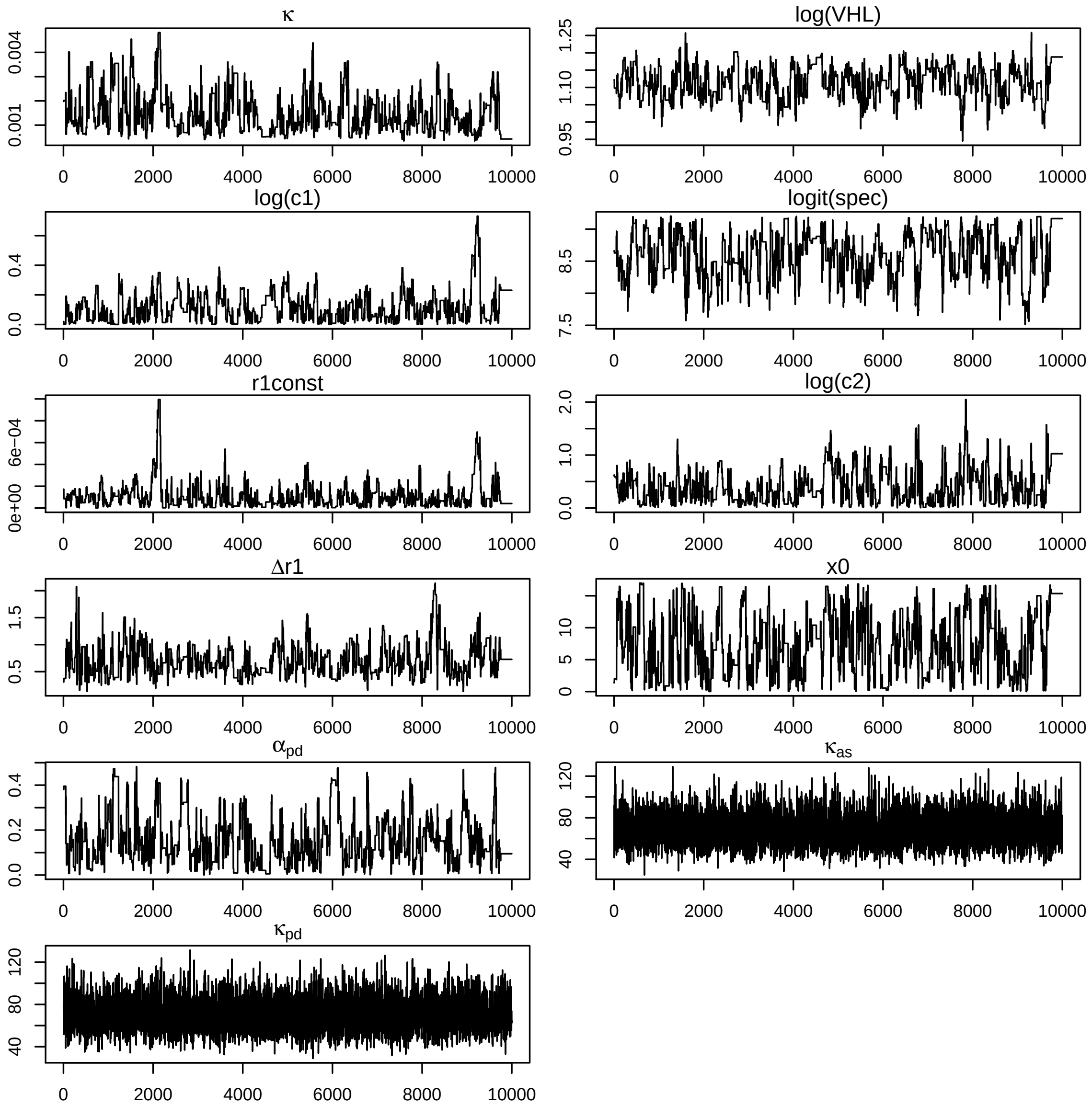

Figure 9: Trace of fitted parameters for Mbaya health zone.
